# A review of the components of exercise prescription for sarcopenic older adults

**DOI:** 10.1007/s41999-022-00693-7

**Published:** 2022-09-02

**Authors:** Prabal Kumar, Shashikiran Umakanth, N. Girish

**Affiliations:** 1 Department of Physiotherapy, Manipal College of Health Professions, Manipal Academy of Higher Education, Manipal, Karnataka India; 2 Department of Medicine, Dr. TMA Pai Hospital, Manipal Academy of Higher Education, Manipal, Karnataka India

**Keywords:** Sarcopenia, Exercise, Older adults, Resistance

## Abstract

**Purpose:**

To synthesize the details of the exercises/exercise program prescribed for the improvement of muscle mass/muscle strength/physical performance among sarcopenic older adults.

**Methods:**

A systematic literature search was conducted in five electronic databases and the details of exercises such as single component or multicomponent exercise program, frequency/week, intensity, duration of the exercise program, type of exercises, progression, adverse events reported, outcome measures used, and whether technology or other educational aids were used to deliver the program were extracted.

**Results:**

A total of 10,045 records were identified and 27 records were included. Resistance exercises were included in all the studies, with the frequency ranging from 1 to 5/week, intensity ranging from 20 to 80% of 1 repetition maximum (RM), or 6–14 points on ratings of perceived exertion (RPE), and duration per session ranging from 20 to 75 min. The intensity of aerobic exercises ranged from 50 to 70% of heart rate max or a level of 7–17 in RPE with a duration ranging from 6 to 30 min per session for 2–5 days/week. For balance exercises, the intensity was mentioned as the level of effort 3 on a scale of 10, and the time duration per session ranged from 5 to 30 min for a frequency of 2/3 per week.

**Conclusion:**

This review synthesized the components of exercise prescription for sarcopenic older adults which would help practitioners and researchers in selecting the frequency, intensity, duration, type, mode, and progression while prescribing exercises.

**Supplementary Information:**

The online version contains supplementary material available at 10.1007/s41999-022-00693-7.

## Key summary points

**Aim:**

To synthesize the details of the exercises prescribed for the improvement of muscle mass/muscle strength/physical performance among sarcopenic older adults.

**Findings:**

Frequency, intensity, duration, type, mode, and progression while prescribing resistance, aerobic, balance, and flexibility exercises for sarcopenic older adults were identified and reported.

**Message:**

A multicomponent exercise program targeting muscle strength, aerobic, balance, and flexibility are recommended and tailored as per the existing fitness level and targeted outcomes for sarcopenic older adults.

**Supplementary Information:**

The online version contains supplementary material available at 10.1007/s41999-022-00693-7.

## Introduction

Sarcopenia among older adults is a disorder that causes a gradual loss of skeletal muscle mass, strength, and deterioration in physical performance [1–3]. The prevalence of sarcopenia ranges from 9 to 10% among community-dwelling older adults, 23–24% among hospitalized people, and 30–50% among residents of long-term care settings, and its incidence increases with age [4, 5]. A plethora of factors contribute to the development of sarcopenia among older adults, including sedentary lifestyle, changes in endocrine function (insulin, testosterone, growth hormone, insulin-like growth factor-1, cortisol), loss of neuromuscular function, an imbalance between muscle protein synthesis and breakdown, insufficient dietary protein intake, and genetic factors [6, 7].

Sarcopenia is concerning as it leads to negative health outcomes in older people, such as falls, frailty, physical restrictions, activity limitations, lower quality of life, and an increased risk of premature death [8–11]. The European Working Group for Sarcopenia in Older People (EWGSOP) and Asian Working Group for Sarcopenia (AWGS) have come up with the diagnostic criteria for sarcopenia among older adults, which considers muscle mass, muscle strength, and physical performance parameters with slight variations in the cutoff values [2, 3]. Among the pharmacological and non-pharmacological management options, the highest evidence exists for a multicomponent program involving exercises as the mainstay [12, 13]. However, there is a conflict among practitioners/clinicians and in literature with regard to the details of the exercise programs, and a review targeting the components of exercises for sarcopenia among older adults is lacking. This literature review aims to synthesize the details of the exercises/exercise program prescribed for the improvement of muscle mass/muscle strength/physical performance among sarcopenic older adults.

## Methods

### Eligibility criteria

The studies were included if they met the criteria: (a) original study, (b) on older adults, (c) including those diagnosed with sarcopenia, (d) with study design such as pilot study, randomized controlled trial, pre–post intervention trial, or longitudinal studies, (e) single-component or multicomponent exercise-based intervention. The studies were excluded if they failed to meet the inclusion criteria and/or: (a) full text was not available, (b) language was other than English, (c) was a single/multiple component intervention in which exercise was not a component, (d) studies providing no extractable data, and (e) patients had secondary sarcopenia due to conditions like COPD, cancer, kidney disease, stroke, Parkinson’s disease, and Alzheimer's disease.

### Data sources and search strategy

A systematic literature search was undertaken in December 2021 using the following electronic databases: PubMed, Scopus, Embase, Cumulated Index to Nursing and Allied Health Literature (CINAHL), and Web of Science. Relevant MeSH terms and Boolean phases were used for the search: “sarcopenia” OR “reduced skeletal muscle mass” OR “muscle mass loss” OR “muscle atrophy” AND “aged” OR “elderly” OR “older adults” OR “older people” AND “physical therapy” OR “rehabilitation programme” OR “therapeutic exercise”  AND “residential care facilities” OR “long-term care facilities” OR “old age homes” without time restrictions and no filters were applied. The complete search strategy for each database is shown in the supporting information (search strategy.pdf).

### Data extraction

Two reviewers, PK and GN, independently searched the literature. The identified studies were imported to Rayyan (Ref. # 366956) software. After resolving the duplicates, two reviewers (PK and GN) conducted title and abstract screening separately. If the study was deemed suitable, it progressed to the retrieval of the full text. If, after a review of the full text, the article was still considered suitable for the analysis, then it progressed to data extraction. Any conflict regarding the article selection was resolved by discussion with the third reviewer, SU. Full-text reading of the identified articles was done by PK, and relevant studies were included in this review matching our inclusion criteria. The data charting form was drafted by PK as per the American College of Sports Medicines’ (ACSM) [14] frequency, intensity, time and type principle (FITT principle) and finalized after a consensus discussion with all the authors. The details of exercises like a single-component or multicomponent exercise program, frequency/week, intensity, duration/session, total duration of the exercise program, type of exercises, progression, adverse events reported, outcome measures used, and whether technology or other educational aids were used to deliver the program were extracted.

## Results

### Search results

**Fig. 1:**
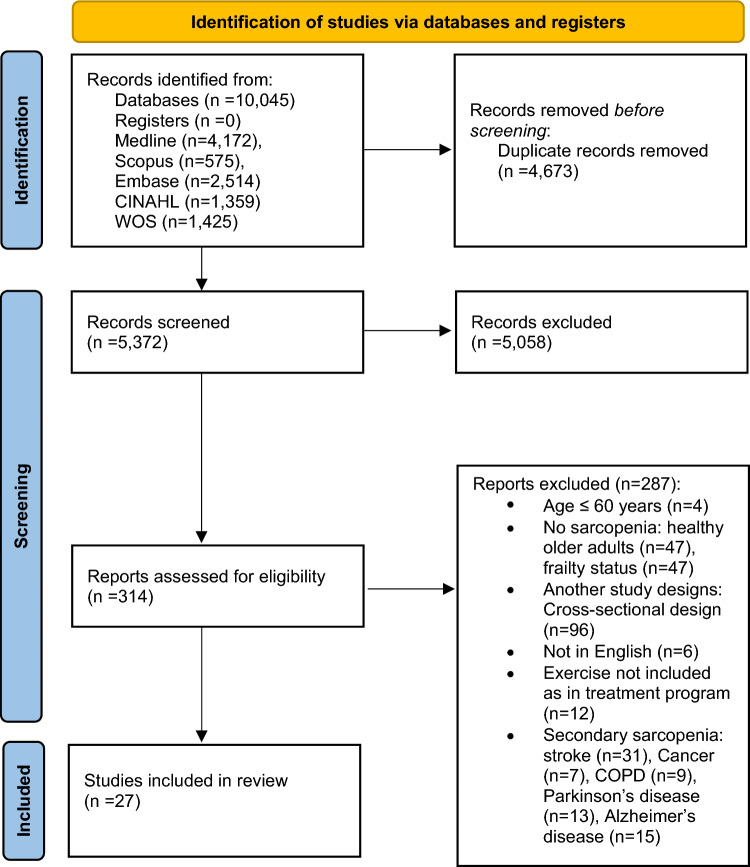
Preferred Reporting Items for Systematic Review and Meta-analysis 2020 (PRISMA 2020) flow diagram

A total of 10,045 studies were identified through database searches. After removing duplicate studies, the titles and abstracts of 5,372 studies were screened. A review of the titles and abstracts yielded 314 relevant studies for full-text screening. Finally, 27 studies met all inclusion criteria and included in this review. A Preferred Reporting Items for Systematic Review and Meta-analysis 2020 (PRISMA 2020) flowchart of the literature search is demonstrated in Fig. 1.

Nine studies used Asian Working Group for Sarcopenia 2014 (AWGS 2014) criteria [15–23], one study each used AWGS 2016 [24] and AWGS 2019 criteria [25], six studies used European Working Group  for Sarcopenia in Older People (EWGSOP 2010) criteria [26–31], one study used EWGSOP 2019 criteria [32], one study used Center for Disease Control and Prevention criteria [33], one used International Working Group on Sarcopenia (IWGS) along with EWGSOP 2010 criteria [34] and seven studies did not report any of the above-mentioned criteria [35–41].

Twenty-seven studies, which used exercise intervention in single- and multicomponent modes, were included in this review. Out of 27, in 16 studies (*n* = 16), the exercise intervention was given as a single component with all studies using resistance exercise [15, 19–23, 25–27, 29, 31, 32, 34, 37, 38, 40], while the remaining 11 studies (*n* = 11) administered multicomponent exercise intervention [16–18, 24, 28, 30, 33, 35, 36, 39, 41]. Among the multicomponent exercise interventions, resistance exercise plus balance exercise were administered in four studies (*n* = 4) [16, 18, 28, 41]; resistance exercise plus aerobic exercise in three studies (*n* = 3) [24, 30, 36], resistance exercise plus aerobic exercise plus balance exercise in one study (*n* = 1) [39]; resistance exercise plus aerobic exercise plus balance exercise plus flexibility exercise in two studies (*n* = 2) [17, 33]; resistance exercise plus balance exercise plus aerobic exercise plus endurance exercise in one study (*n* = 1) [35]. In 25 studies (*n* = 25), a supervised exercise program [15, 17, 18, 20–41], in one study (*n* = 1) an unsupervised exercise program [16], and in one study, (*n* = 1) an initial training was administered and then participants performed exercise unsupervised [19].

The details of the exercise program are summarized in Tables 1, 2, 3, and 4 for resistance exercises, aerobic and endurance exercises, balance exercises, and stretching exercises, respectively.

Of the 27 studies on resistance exercises, 9 studies (*n* = 9) used repetition maximum (RM) criteria [18, 25, 27, 29, 30, 32, 36, 37, 40], whereas 10 studies (*n* = 10) used rating of perceived exertion (RPE) [15, 19, 21, 23, 26, 28, 31, 38, 39, 41] criteria to quantify the intensity of resistance training. One study each (*n* = 1) used OMNI-RES scale (OMNI-Rating of perceived Exertion scale) [34], heart rate reserve criteria [20] or coach-adjusted resistance criteria [24], and maximal theoretical force [33] while four studies (*n* = 4) [16, 17, 22, 35] did not report the method used for intensity measurement. The percentage of 1RM, which ranged from 20 to 80% of 1RM, was used by all the nine studies that used RM criteria, and of the 10 studies (*n* = 10) that used RPE, it ranged from 6 to 14 points. The frequency/week of resistance training ranged from 1 to 5/week. Eight studies (*n* = 8) administered resistance training two/week [15, 18, 28–30, 32, 33, 41], 14 studies (*n* = 14) three/week [20–27, 31, 34–38], 1 study (*n* = 1) each one [40] and five per week [19], respectively. A total of three studies (*n* = 3) did not report the frequency of resistance training [16, 17, 39]. The duration per session ranged from a minimum of 20 to a maximum of 75 min and the total duration of the intervention program ranged from 6 to 32 weeks. The progression of resistance exercise has been reported in 22 studies (*n* = 22), among which 9 (*n* = 9) [18, 25, 27, 28, 30, 32, 33, 36, 40] reported progression in terms of the number of sets, repetition per set, and intensity, either RM or RPE, 2 studies (*n* = 2) [34, 37] reported progress in resistance exercise using OMNI, 10 studies (*n* = 10) [15, 17, 19, 21, 22, 26, 29, 31, 38, 41] reported progression with respect to the loading method, and 1 study (*n* = 1) [20] reported in terms of duration/session. A total of five studies (*n* = 5) [16, 23, 24, 35, 39] did not report the progression of resistance training.

**Table 1:** Prescription components for resistance exercises (*n* = 27)

Author (s) details, study design and settings	Participants (number and age)	Diagnostic criteria	Single/multicomponent program	Frequency/week	Intensity	Time/session	Total duration (weeks)
Laddu et al. [32], Pilot study	IG (*n* = 60) CG (*n* = 30)	EWGSOP 2	Single component	2/week	40–80% 1RM	Protocol	12
Seo et al. [34], RCT and Community dwelling	IG-12 (Age 70.3 ± 5.38) CG-10 (Age 72.9 ± 4.75)	IWGS and EWGSOP	Single component	3/week	Intensity OMNI Scale/Colour; week 1-2: 4/yellow; week 3–4: 5/yellow; week 5–8: 6/yellow; week 9–12: 7/yellow; week 13–16: 8/yellow	Warm-up (stretching): 5 min; Resistance exercise: 50 min; cool down (Stretching): 5 min	16
Kuptniratsaikul et al. [19], Prospective longitudinal clinical trial and community dwelling	*n* = 89 (Age 69.4 ± 6.3)	AWGS	Single component	3–5/week	Tolerable	30 min	24
Chang et al. [30], RCT	Early intervention 29 (age 74.3 ± 5.8) Delayed intervention 29 (age 75.7 ± 5.9)	EWGSOP	Multicomponent	At OPD 2/week Home-based 5 or more days/week	40% 1RM	At OPD Warm-up 10 min Resistance exercise 25 min Cool-down 10 min Home-based exercise program 30 min/day (with total of 150 min per week)	12
Osuka et al. [15], RCT, community-dwelling older adults	Exs + HMB *N* = 36, Exs + placebo *n* = 37, education + HMB *n* = 36, education + placebo *n* = 35	AWGS	Single component	2/week	< 12 RPE	60 min	12
Chiang et al. [23], RCT, nursing home residents	CG (only exercise)12(Age 84.67 ± 7.5) Milk + exercise 12 (Age—85.25 ± 5.38) Soymilk + exercise 12 (age 85 ± 5.62)	AWGS	Single component	3/week	Mild	30 min	12 Weeks
Caballero-García et al. [39], placebo-controlled trial	*N* = 44 Placebo group 22 Citrulline-malate supplemented group 22 Avg age M 64.8 ± 3.6, F 65.4 ± 4.4	NR	Multicomponent	NR	Level of effort 8 reps	20 min	6
Chen et al. [25], RCT, community dwelling	*n* = 51	AWGS	Single component	3/week	LRT-BFR: 20–30% 1RM; CRT: 60–70% 1RM	LRT-BFR: 30 s interval between sets; CRT: 60 s interval between sets	12
Moghadam et al. [36]	ET + RT = 10 RT + ET = 10 CG = 10	NR	Multicomponent	3/week	40–75% 1RM	2 min interval between sets	8
Makizako et al. [17], RCT and community dwelling	IG: *n* = 33(Avg age-74.1 ± 6.6, CG: *n* = 34(Avg age 75.8 ± 7.3)	AWGS	Multicomponent	NR	NR	25–30 min	12
Liang et al. [18], RCT and post-acute care unit	IG (*n* = 30), CG (*n* = 29) Avg. age 87.3 ± 5.4 years	AWGS	Multicomponent	2/week	70–80% 1RM	20 min training	12
Chow et al. [22], RCT and community dwelling	EXS + HMB: *n* = 48, CG: *n* = 48 and vibration + HMB: *n* = 48	AWGS	Single component	3/week	NR	30 min	12
Letieri et al. [37], RCT and community dwelling	LI-BFR (*n* = 11) (69.40 ± 5.73 years), CG (*n* = 12) (69.00 ± 6.39 years)	NR	Single component	3/week	20–30% 1RM	20 min	16
Jung et al. [20], community dwelling	EG: *n* = 13 (75.0 ± 3.9 years),CG: *n* = 13 (74.9 ± 5.2 years)	AWGS	Single component	3/week	60–80% HRR	75 min	12
Martin Del Campo Cerventes et al. [31], longitudinal intervention study and nursing homes	*n* = 19 (Avg. age 77.7 ± 8.9 years)	EWGSOP	Single component	3/week	Moderate–high intensity	NR	12
Vikberg et al. [26], RCT and community dwelling	IG: *n* = 31(70.0 ± 0.29 years), CG *n* = 34 (70.9 ± 0.28)	EWGSOP	Single component	3/week	Moderate–high RT intensity Borg 6–7 of 10 maximum	45 min with group of < 12 participants	10
Granic et al. [29], Pilot study and community-dwelling older adults	Protocol: *n* = 30	EWGSOP	Single component	2/week	70–79% 1RM	45–60 min per session	6
Zhu et al. [24], RCT and community dwelling	RT: *n* = 40 (74.5 ± 7.1) E + nutrition: *n* = 36(74.8 ± 6.9) WL: *n* = 37(72.2 ± 6.6)	AWGS	Multicomponent	3/week (twice group exercise session and one home exercise session)	Closely monitored and adjusted by the coach	5–10 min warm-up and cool-down Resistance exercise 20–30 min per session	12
Jeon et al. [21], community-dwelling elderly	*n* = 30 (Age 73.8 ± 5.9 years)	AWGS	Single component	3/week	Borg scale	30 min	6
Ude Viana et al. [27], quasi-experimental study and community-dwelling older women	*n* = 18 (Avg Age: 75.11 ± 7.19 Years)	EWGSOP	Single component	3/week	75% 1RM	40 min	12
Najafi et al. [35], RCT and nursing home	IG: *n* = 35 CG: *n* = 28 (Avg. Age—72.5 ± 7.0)	NR	Multicomponent	3/week	NR	IG—fun physical activity20 min/session CG regular physical activity 20 min/session	8
Hassan et al. [28], pilot study and nursing care facilities	EX: *n* = 18, CG: *n* = 21 (Avg age: 85.9 ± 7.5 years)	EWGSOP	Multicomponent	2/week	12–14 on Borg scale	60 min	24
Hong et al. [38], RCT and community-dwelling senior citizens	23 elderly, Tele—Tele exs: *n* = 9 (82.2 ± 5.6 years), CG: *n* = 11 (81.5 ± 4.4 years)	NR	Single component	3/week	Somewhat hard (RPE 13–14) and hard (RPE 15–16)	10–30 min	12
Maruya et al. [16], community dwelling	IG: *n* = 26 (69.2 ± 5.6 years); CG: *n* = 14 (68.5 ± 6.2 years)	AWGS	Multicomponent	NR	NR	20–30 min	24
Bellomo et al. [33], RCT	Gsm: 10, RT: 10, Vam: 10, CG: 10 (Avg age—70.9 ± 5.2)	Center for disease control and prevention	Multicomponent	2/week	60–85% FMT	NR	12
Sousa et al. [40]	EG: *n* = 16 (68.5 3.5 years. CG: *n* = 17 (67.0 ± 5.8 years)	NR	Single component	1/week	65–75% 1RM	NR	32
Kim et al. [41], RCT, urban and community	E + AAS: *n* = 38 (79.5 ± 2.9 years), E: *n* = 39(79.0 ± 2.9 years), AAS: *n* = 39(79.2 ± 2.8 years), HE: *n* = 39 (78.7 ± 2.8 years)	NR	Multicomponent	2/week	12–14 RPE	30 min	12

Author (s) details, study design and settings	Type of exercise(s)	Mode of exercise(s)	Progression	Outcome measure(s)	Adverse event(s)	Educational aids/technology used	Finding(s)
Laddu et al. [32], Pilot study	Progressive resistance exercise	Upper- and lower extremity exercises: Chest press, Seated Leg press, seated latissimus pull-down, knee/leg extension, shoulder press, leg curls, and calf-raises	Week 1- 1–2 sets, 10–15 reps, 40–50% 1RM Week 2—2 sets,8–12 reps, 60–65% 1RM Week 3–12 3 sets, 8–12 reps, 60–65% 1-RM/70–75% 1RM/80% 1-RM/80% 1-RM	Primary outcome: SBP; Secondary outcome: Lipid profile, insulin resistance (HOMA IR) and inflammation (IL-6), HGS, 6-m gait speed usual walking speed, five time sit to stand, 3stage standing balance, TUG, SPPB	Protocol	NR	Protocol
Seo et al. [34], RCT and Community dwelling	Elastic band Resistance exercise program	Warm-up stretching and walking Resistance exercise (Upper body)—Shoulder press, front raise, lateral raise, biceps curl, triceps extension, kick back, crunch, bent row over, seated row, back extension in prone, push up beginner Resistance exercise (Lower body)—Squat, lunge, lying leg abduction, leg kick back, pelvic tilt, leg raise, toe, and heel raise Cool down—static stretching	RT: Training load was increased by progressive overload and the OMNI resistance for active muscle scale (OMNI-RES AM, 0-extremely easy to 10-extremely hard)	Body composition (FFM, BF%, Fat mass,) (DXA), Functional fitness (senior fitness battery test (walking 2-min step test, chair stand, chair sit and reach, 2.4 m up and go and arm curl), grip strength, gait speed), Mid-thigh composition (CT scan), Maximal Isometric muscle strength (Isokinetic dynamometer), Biochemical markers (ELISA)	NR	NR	16 weeks of resistance training using body weight-based training and elastic bands significantly improves muscle quality and functional fitness in sarcopenic older women. However, it remains unclear whether our training protocol affects muscle growth factors
Kuptniratsaikul et al. [19], Prospective longitudinal clinical trial and community dwelling	Home-based resistance exercise program	Intrinsic hand Shoulder muscle Pectorals Abdominals Back extensors Hip flexion/extension Ankle plantar flexion/dorsi flexion	1–3 Months—Body weight Later 1 kg dumbbell + body weight	ASM (BIA), grip strength (JAMAR HHD), gait speed (6-m test), functional reach	Muscle pain, Joint pain, Fatigue No SAE	CD, brochure, Logbook	24-week simple home-based resistance exercise program significantly improved all main outcomes with low adverse events, and most participants continued the program after the end of the intervention
Chang et al. [30], RCT	Resistance exercise and Aerobic exercise	Warm-up stretching trunk and all limbs plus stationary bicycle Exercise—leg press, leg extension, leg curl Cool down—bicycle	Starting with 3 sets, 10 reps, 40% 1RM Progress to 80% 1RM	Physical performance: Grip strength (Baseline Hydraulic HHD), Gait speed (5-m walk test), 30 s chair stand test, 2-min step test; Body composition: Bone mineral content, Fat mass and lean body mass (DEXA)	No AE	Digital versatile disc, handbook	Significant difference in LE lean mass between baseline and both follow up in early intervention group Significant difference in total lean mass between baseline and 1st follow up in delayed intervention group Both group significant improvement in grip strength, 2-min step test from baseline to 2nd follow up
Osuka et al. [15], RCT, community-dwelling older adults	Resistance exercise	Chair based RT: Knee ext, toe raise, heel raise, knee lift, squats, lateral leg raise, and hip add, using a rubber balls Knee lift and heel raise exercises were performed progressively from a seated position to a standing position Elastic band RT: Arm rowing, knee lift, and hip Adduction, Knee extension, heel raise, knee lift, and lateral leg exercises were provided using ankle weights of 0.5, 0.75, 1.0, or 1.5 kg based on the participant’s physical condition In the last 4 week of the intervention**,** machine-based RT, including arm rowing, leg extension, hip adduction, knee extension, and trunk flexion	Exercise including chair-based (week 1–12), elastic band (week 5–7), ankle weight (week 7–12) and machine-based RT (week 9–12) All exercises 1–3 sets, 8–10 reps with gradual loading	Primary outcome: Muscle mass(BIA), Secondary outcome: muscle strength(Smedley type HHD), physical performance, functional capacity, blood markers, habitual dietary intake, and habitual physical activity levels	No exercise related AE observed	NR	HMB additively improved gait performance with negligible benefit and provided no enhancements in the effects of exercise on other outcomes. Exercise appeared to be the only effective intervention to improve outcomes in older women with low muscle mass
Chiang et al. [23], RCT, nursing home residents	Resistance exercise training program	Chair exercise, resistance exercise with sandbags and elastic bands	NR	Anthropometric data: fat mass and lean mass (DEXA), Sarcopenic indices: muscle mass, body fat and ASMI (BIA), HGS (Smedley Dynamometer), GS: 6-m walk test	NR	NR	Mild resistance exercise for 12 weeks improved the calf circumference and gait speed; in addition, mild resistance exercise combined with milk or soy milk (400 mL/day) supplementation also increased HG and CC in very old nursing home residents with sarcopenia. No obvious effects were found in the muscle mass of very old individuals with sarcopenia
Caballero-García et al. [39], placebo-controlled trial	Aerobic resistance Aerobic endurance Balance	Aerobic resistance—Overload exercises, with balls, dumbbells, elastic bands, steps	NR	6 min test (endurance) on 400-m track, HGS (JAMAR digital Dynamometer), Gait speed (4-m test), Squat, SPPB, Balance (Standing, semi tandem, tandem stand)	NR	NR	No significant difference in the outcome measures between placebo and intervention group
Chen et al. [25], RCT, community dwelling	Low resistance training-Blood flow restriction and Conventional Resistance training	Upper limb exercises (elbow extension and elbow flexion), followed by lower limb exercises (leg press and knee extension	RT:Week 1–4: LRT-BFR: 3 sets/30–15-15 reps, 20% 1RM, CRT: 3 sets/15 reps, 60% 1RM Week 5–8: LRT-BFR: 3 sets/30–15-15 reps, 25% 1RM, CRT: 3 sets/12 reps, 65% 1RM; Week 9–12: LRT-BFR: 3sets/30–15-15 reps, 30% 1RM, CRT: 3 sets/10 reps, 70% 1RM	Primary outcome: lower limb muscle strength (estimated 1RM of knee extension) Secondary outcomes: Body composition (BIA), Hand grip strength (HHD), Muscle performance (SPPB), Pulmonary function (PFT), Blood biomarker (ELISA) and CVD risk factors and Health-related quality of life (SF-36)	Protocol	Protocol	Protocol
Moghadam et al. [36]	Resistance exercise + aerobic exercise	Leg extension, leg curl, bench press, lateral pulldown, lateral raise, and abdominal crunch	RT: Week 1–2: 14–16 reps, 2 sets, 40–45% 1RM Week 3–4: 12–14 reps, 2 sets, 50–55%1RM Week 5–6: 10–12 reps, 3 sets, 60–65%1RM Week 7–8: 8–10 reps, 3 sets, 70–75% 1RM	Body composition (BIA); Performance testing: Strength (1RM), Power (30-s vintage test on cycle ergometer, cardiorespiratory fitness (modified Bruce protocol for VO2 max)	NR	Diet analysis plus version 10 was used to record data	8-week of CT intervention increased circulating SC related markers, body composition, enhanced muscular power, and VO2 max in older sarcopenic participants, regardless of the order of ET and RT. However, performing ET before RT may be more effective at enhancing Myf5 and Pax7, as well as improving both lower and upper body power
Makizako et al. [17], RCT and community dwelling	Resistance exercise + aerobic exercise + balance exercise + flexibility	(1) knee ext (2) hip Flex, (3) hip IR, (4) elbow flexion and shoulder abduction, (5) elbow flexion and trunk rotation, (6) hip ext, (7) knee flex, (8) hip abd, and (9) squat	RT: Week 1–2: low load (own body weight), progressive resistance with resistance band with five resistance level every two weeks after assessment of strength that is 12–14 RPE on 10 RM of knee extension. For each exercise 10 reps	Physical performance: Grip strength (HHD), Gait speed (6-m test), 5-Chair stand test, TUG and Muscle CSA and volume (MRI)	No AE reported	Infrared timer for gait speed assessment (Outcome measure) Booklet	12 week multicomponent exercise program with progressive resistance training generally improves physical function in CDOA with sarcopenia or pre-sarcopenia. However, it is unclear whether effective in increasing muscle mass
Liang et al. [18], RCT and post-acute care unit	Resistance exercise + balance exercise	Leg press, leg extension and flexion, leg abduction and adduction, chest press, and seated row	Resistance training: 3 sets of 8–12 reps with 2 min rest in between, load adjusted after 13thsession	Primary outcomes: Activities of daily living (Barthel index) and number of fallers; Secondary outcomes: SPPB, 4-m gait speed, HGS(Digital grip dynamometer), Berg balance, TUG, and any adverse events	NR	NR	Compared with resistance exercise, the mixed exercise program (Balance plus resistance exercise) appeared to have improved the ADL, strength, and physical performance in older sarcopenic patient in post-acute care settings
Chow et al. [22], RCT and community dwelling	Resistance exercise (Group 1) and Vibration exercise (Group 2)	Upper and lower body muscle groups including both hand and knee extensor muscles	Resistance training: Elastic band strength progressively increased from 1.3 kg to 2.1 kg (Yellow to green) based on multiple RM described as fatigue reaching by 8 reps of stretching	Primary outcome: Knee extension strength; Secondary outcome: HGS, GS, MM, Balancing activity, TUG test, SARC-F, SF-36, Food frequency questionnaire, activity tracker (steps)	Protocol	Wrist worn activity tracker to record daily activity	Protocol
Letieri et al. [37], RCT and community dwelling	Resistance training with blood flow restriction	Leg squat, leg press, leg extension/flexion and stand plantar flexion	OMNI scale	Body fat % (BIA), Functional capacity: Chair stand, Arm curl, Sit and reach, TUG, Back scratch, and 6 min’ walk test, HGS (Dynamometer), Appendicular muscle mass (using equation)	NR	NR	Exercise conducted with BFR associated with low intensity resulted in a significant improvement in the functional capacity of elderly women after 16 weeks. Despite the significant results the intervention period was not sufficient to reverse the pre-sarcopenia condition in elderly women
Jung et al. [20], community dwelling	Resistance exercise	Walking in place, shoulder press and squat, twist dash, lunge, jumping jacks, kick back, push up, crunch, hip bridge, and bird dog	Week 1–2: 25 min, Week 3–8: 40 min, Week 9–12: 55 min	Body composition (BIA), Balance (Posturomed), Muscular function (Isokinetic dynamometer), Pulmonary function(FVC, Forced expiratory volume in 1 s, forced expiratory flow 25–75%), 10-m walk (s)	NR	NR	Circuit exercise training improves muscle mass and strength, body composition, balance, and pulmonary function in women with sarcopenia
Martin Del Campo Cerventes et al. [31], longitudinal intervention study and nursing homes	Resistance exercise	Resistance training scheme was developed based on the recommendation of the American College of Sports Medicine	2–3 sets, 8–12 reps (1–2 months), 2–3 sets, 15 reps (3^rd^ month), Dumbbells of 0.5, 1 and 3 kg as well as elastic bands of three resistance (medium, strong, and extra strong)	Muscle strength: HGS (SMEDLEY Dynamometer) and physical function: SPPB (balance, gait speed, chair stand), Muscle mass and fat mass (BIA)	Fall	NR	The resistance training program improve the functionality (muscle strength and physical performance), with the benefit of the decrease in severe sarcopenia
Vikberg et al. [26], RCT and community dwelling	Resistance exercise	More focus on Lower limb strengthening	Week 1:body weight and suspension band, 2 sets,12 reps; Week 2–4: 3 sets, 10 reps, intensity increased CR-10 scores of 6–7; Week 5–7: 4 sets,10 reps; Week 8–10: power training	Primary outcome: SPPB; Secondary outcome: TUG, Chair sit-stand time, lean body mass (Lunar iDXA device) and fat mass (iDXA scan), HGS (JAMAR Hydraulic HHD)	Pain in shoulder, vertigo, delayed onset muscle soreness	Supplementary video to describe exercises	The main finding of this intervention study is that an easy -to-use, functional resistance training program was effective in maintaining functional strength and increasing muscle mass in older adults with pre-sarcopenia
Granic et al. [29], Pilot study and community-dwelling older adults	Resistance exercise	Leg press, leg curl, seated row, chest press	Intensity monitored using CR-100 scale	Primary: Feasibility, applicability dosage and duration of intervention, compliance, adverse health effects, response rates to questionnaire; Secondary: SPPB (balance, 4 m gait speed, 5 chair stand), Muscle mass (BIA), Grip strength (JAMAR HHD), SF-12 Health survey, Barthel index	Protocol	Protocol	Protocol
Zhu et al. [24], RCT and community dwelling	Resistance exercise and aerobic exercise	Chair based resistance exercises using Thera band	NR	Primary outcome: Change in gait speed over 12 weeks (6-m walk test); Secondary outcome: Muscle strength, muscle power, body composition, health related QOL(SF-36), physical activity scale for the elderly, instrumental activities of daily living and cardiorespiratory fitness; tertiary outcome: to follow till 24 weeks	4 AE and 12 SAE but none related to prescribed intervention	NR	The exercise program with and without nutrition supplementation had no significant effect on the primary outcome of gait speed but improved the secondary outcomes of strength, and the 5 CST in community-dwelling Chinese sarcopenic older adults
Jeon et al. [21], community-dwelling elderly	Resistance exercise	Mechanically-assisted squat device program	Squat exercise: Week 1–3: exs program for 30 min at RPE 12–14, After week 3: emphasis on RPE 14–16 for 30 min; 6–7 rotation of sitting to supine to tilt positions were performed	Pulmonary function test (Micro Lab ML3500 MK8 platform): FVC, FEV1sec, MIP and MEP; Knee extensor strength (HHD), Grip strength (Handheld digital grip dynamometer), 3 min walk test, Whole body lean mass (DEXA)	NR	NR	Mechanically assisted squat exercises improved muscle function, including the strength of both knee extension and hand grip, in subject with or without sarcopenia. Leg lean mass and SM was increased in subject without sarcopenia also improve FVC. A prospective RCT exploring effects of mechanically assisted squat exercise by subjects with sarcopenia is essential to definitively confirm the efficacy
Ude Viana et al. [27], quasi-experimental study and community-dwelling older women	Progressive Resistance Training Program	Knee extension/flexion, hip extension, flexion, abd and bridge hip + hip abd using a ball and semi-squat. Ankle weights were used to perform the exercises with 1 min interval between the three sets of 12 repetitions each	Resistance training: 3 sets of 12 reps each exercise with 1 min interval between sets, load reassessed every 2 weeks	Muscle strength of Knee extensors (Isokinetic dynamometry), Muscle mass (DEXA), Functional performance (SPPB)	NR	NR	The progressive resistance training program was able to counteract losses on muscle mass, strength, physical performance in community-dwelling sarcopenic older adults and this kind of exercise could be used safely to avoid the negative impact of the loss of strength and muscle mass on sarcopenia
Najafi et al. [35], RCT and nursing home	Strength, walking, balance, endurance activities	Regular PA include—daily walking for 30 min plus stretching Fun PA group—strength, balance, endurance, and walking activities (in the form of rotational movement of hands with plastic balls (also k/a beach balls), catch-a-colour rockets, wands, Audubon bird and stretch bands)	NR	Balance (BBS), 6 min walk distance (6-MWT), Muscle strength (Dynamometer)	NR	NR	Fun PA reduces sarcopenic progression through improving balance, increasing distance walked, and strengthening muscles
Hassan et al. [28], pilot study and nursing care facilities	Resistance and balance training	Elbow and shoulder extension (dip), leg press, knee ext/flex, hip abd/add, abdominal curl and back extension	RT: 2-week conditioning following 2–3 sets per exercise at intensity they could do 10–15 times with RPE 12–14, progression increasing load if complete 3 sets of 10 reps/set or by increasing with 3 sets of 15 reps	Number of falls, QOL, functional performance (SPPB), falls efficacy and cognitive wellbeing	No adverse event	NR	Resistance and balance exercise has positive benefits for older adults residing in nursing care facilities which may transfer to reduce disability and sarcopenia transition, but more work is needed to ensure improved program uptake among residents
Hong et al. [38], RCT and community-dwelling senior citizens	Resistance exercise	Bicep curls, triceps curls, front raises, leg raises, leg curls, leg extensions, squats, and calf raises	RT: Week 1–4: no weight, Week 5–8: 1 kg Dumbbell, Week 9–12: 2 kg Dumbbell, progressively increased by about 2 steps every 4 weeks from RPE 11–15, 3 sets of 8–10 reps, interval between each set 1 min The total exercise time was progressively increased by 20 to 40 min during the intervention period	Body composition (BF%, UL and LL muscle mass and appendicular lean soft tissue) DEXA, Functional fitness: senior fitness test	NR	Skype	Tele-exercise based on video conferencing would enable real time interactions between exercise instructors and elderly adults and could prove to be a new scientific, safe, and effective intervention method for preventing or improving sarcopenia, thus enhancing QOL among the elderly population
Maruya et al. [16], community dwelling	Home-based lower extremity Resistance and balance exercise program	Lower limb resistance exercises and balance exercises were used: squats, single-leg standing, and heel raises	NR	Body composition (SMI,BMI and body fat %) using BIA, Self-reported QOL (EQ-5D, GLFS-25), Physical function (HGS, duration of single leg stand, comfortable and maximum walking speed, and knee extension strength (Handheld Dynamometer)	NR	Guide book	A 6-month home exercise program, combining walking and resistance LL exercise, was effective in improving maximum walking speed and muscle strength in individual, in more than 60 years old with pre sarcopenia and sarcopenia
Bellomo et al. [33], RCT	Global sensori motor: Aerobic, balance and flexibility training; Resistance training; Vibratory mechanical-acoustic focal therapy	Leg press and leg extension	RT: 1–4 weeks: 3 sets of 12 reps with 60–70% FMT; 5–8 weeks: 3 sets of 10 reps with 75–80% FMT; 9–12 weeks: 3 sets of 6–8 reps with 80–85% FMT	Maximal isometric test (Knee extension machine); Gait analysis: Length of half step (cm), Sway area (mm^2^), Ellipse surface (mm^2^)(Pedobarographic platform)	NR	NR	All the training programs implemented in the present investigation increase muscle strength. In addition, sensorimotor and vibrational training intervention aims to transfer these peripheral gains to the functional and more complex task of balance, in order to reduce the risk of falls
Sousa et al. [40]	Resistance exercise	Bench press, leg press, latissimus dorsi pull-down, leg extension, military press, leg curl, and arm curl)	3 sets of 8–12 reps	Dry lean mass (kg), BF% (BIA), muscle strength: 30 s chair stand and arm curl test, maximum strength (1RM)	NR	NR	A once-weekly RT session improves muscle strength and induces beneficial effects in the functional fitness of older adults. The results of the present study suggest that a once weekly session of RT is enough to prevent sarcopenia
Kim et al. [41], RCT, urban and community	Resistance exercise, balance	*Ankle weight exercise***—** *Seated* knee flexion and extension *Standing* knee flexion and extensions *Exercise using resistance bands—* *Lower body***—**leg extension and hip flexion *Upper body***—**double arm pull downs and biceps curls	Resistance exercise: weights of 0.50, 0.75, 1.00 and 1.50 were prepared and used in accordance with each participants strength level as the resistance progressively increased, each exercise 8 reps	Body composition (BIA); functional fitness parameter (muscle strength and walking ability)	NR	NR	Exercise and AAS together may be effective in enhancing not only muscle strength, but also combined variables of muscle mass and walking speed and of muscle mass and strength in sarcopenic women

IG, intervention group; CG, control group; EWGSOP, European Working Group in Sarcopenia for Older People; RM, repetition maximum; SBP, systolic blood pressure; IL, interleukin; TUG, timed up go; SPPB, short physical performance battery; IWGS, International Working Group in Sarcopenia; FFM, fat free mass; BF, Body fat; DXA, dual energy X-ray absorptiometry; ELISA, enzyme linked immunosorbent assay; AWGS, Asian working group for Sarcopenia; ASM, appendicula skeletal mass; BIA, bioimpedance analyzer; HHD, hand held dynamometer; RPE, rating of perceived exertion, RT; resistance training; AE, Adverse events; HMB, hydroxy methyl butyrate; HGS, hand grip strength; GS, gait speed; LRT-BFR, low resistance training Blood flow restriction; BMI, body mass index; QOL, quality of life, FMT, maximal theoretical force

**Table 2:** Prescription components for Aerobic and Endurance exercises (*n* = 7)

Author (s) details, Study design and settings	Participants (number and age)	Diagnostic criteria	Single/multicomponent program	Frequency/week	Intensity	Time/session	Total duration (weeks)	Type of exercise(s)	Mode of exercise(s)	Progression	Outcome measure(s)	Adverse event(s)	Educational aids/technology used	Finding(s)
Chang et al. [30], RCT and physiotherapy OPD and Home based	Early Intervention—29 (age—74.3 ± 5.8) Delayed intervention—29 (age—75.7 ± 5.9)	EWGSOP	Multicomponent	5 days/week	Moderate intensity	150 min/week	12	Resistance exercise and aerobic	Walking	NR	Physical performance: grip strength (hydraulic HHD), gait speed (5-m walk test), 30 s chair stand test, 2-min step test; body composition: fat mass and lean body mass (DEXA)	NR	Digital versatile disc, handbook	Early exercise and nutritional intervention may be helpful in an earlier restoration of lower extremity muscle mass but not physical function in sarcopenic elders. When designing a rehabilitation program for patient with sarcopenia, RT with nutrition support can be prescribed first for the rapid enlargement of the muscle volume, and structuralized home-based exercise can be administered subsequently to preserve the prior intervention effect
Caballero-García et al. [39], Placebo controlled trial and Health centers	*N* = 44 Placebo group—22 Citrulline-malate supplemented group—22 Avg age—M 64.8 ± 3.6, F—65.4 ± 4.4	NR	Multicomponent	NR	Level of effort 7	10 min/session	6	Aerobic resistance Aerobic endurance Balance	Aerobic endurance—walking, Slow running	NR	6 min test (endurance) on 400-m track, HGS (JAMAR digital Dynamometer, Gait speed (4-m test), Squat, SPPB, Balance (Standing, semi tandem, tandem stand)	NR	NR	No significant difference in the outcome measures between placebo and intervention group
Moghadam et al. [36]	ET + RT = 10 RT + ET = 10 CG = 10	NR	Multicomponent	3/week	55–70% HR max (11–17 RPE Borg scale)	15–30 min/session	8	Resistance + endurance exercise	Cycling on a fixed-speed cycle ergometer	ET: week 1–4: 15 min, 55% HRmax, 11 RPE; week 5–6: 25 min, 65% HRmax, 15 RPE; week 7–8: 30 min, 70% HRmax, 17 RPE	Body composition (BIA); performance testing: strength (1RM), power (30-s vintage test on cycle ergometer, cardiorespiratory fitness (modified Bruce protocol for VO2 max)	NR	Diet analysis plus version 10 was used to record data	8-week of CT intervention increased circulating SC related markers, body composition, enhanced muscular power, and VO2 max in older sarcopenic participants, regardless of the order of ET and RT. However, performing ET before RT may be more effective at enhancing Myf5 and Pax7, as well as improving both lower and upper body power
Makizako et al. [17], RCT and Community dwelling	IG: *n* = 33(Avg age-74.1 ± 6.6, CG: *n* = 34(Avg age-75.8 ± 7.3)	AWGS	Multicomponent	NR	NR	20–25 min of balance and aerobic, 6 min stepping exercise	12	Resistance training, Balance, flexibility, and aerobic exercises	Anterior–posterior or lateral stepping repetitions for six minutes	NR	Physical performance: Grip strength (HHD), Gait speed (6-m test), 5-Chair stand test, TUG and Muscle CSA and volume (MRI)	No AE	Infrared timer for gait speed assessment (Outcome measure) Booklet	12 week multicomponent exercise program with progressive resistance training generally improves physical function in CDOA with sarcopenia or pre-sarcopenia. However, it is unclear whether effective in increasing muscle mass
Zhu et al. [24], RCT and Community dwelling	RT: *n* = 40 (74.5 ± 7.1) E + nutrition: *n* = 36(74.8 ± 6.9) WL: *n* = 37(72.2 ± 6.6)	AWGS	Multicomponent	3/week (2/week group exercise and 1 home exercise)	NR	20 min per session	12	Resistance exercise and aerobic exercise	Aerobic exercises	NR	Primary outcome: Change in gait speed over 12 weeks (6-m walk test); Secondary outcome: Muscle strength, muscle power, body composition, health related QOL(SF-36), physical activity scale for the elderly, instrumental activities of daily living and cardiorespiratory fitness; tertiary outcome: to follow till 24 weeks	4 AE and 12 SAE But none were related to prescribed intervention	NR	The exercise program with and without nutrition supplementation had no significant effect on the primary outcome of gait speed but improved the secondary outcomes of strength, and the 5 CST in community-dwelling Chinese sarcopenic older adults
Najafi et al. [35], RCT and Nursing home	IG: *n* = 35 CG: *n* = 28 (Avg. Age—72.5 ± 7.0)	NR	Multicomponent	3/week	NR	20 min per session	8	Strength, walking, balance, endurance activities	Regular PA include—daily walking for 30 min plus stretching Fun PA group—strength, balance, endurance, and walking activities (in the form of rotational movement of hands with plastic balls (also k/a beach balls), catch-a-colour rockets, wands, Audubon bird and stretch bands)	NR	Balance (BBS), 6 min walk test, Muscle strength (Dynamometer)	NR	NR	Fun PA reduces sarcopenic progression through improving balance, increasing distance walked, and strengthening muscles
Bellomo et al. [33], RCT	Gsm: 10, RT: 10, Vam: 10, CG: 10 (Avg age—70.9 ± 5.2)	Centers for disease control and prevention	Multicomponent	2/week; 5 min warm-up	60% HR max	NR	12	Global sensori motor: Aerobic, balance and flexibility training; Resistance training; Vibratory mechanical-acoustic focal therapy	For warm-up in Global sensorimotor group—cycle ergometer For warm-up in resistance training group-stationary bicycle	NR	Maximal isometric test (Knee extension machine); gait analysis: length of half step (cm), Sway area (mm^2^), ellipse surface (mm^2^)(Pedobarographic platform)	NR	NR	All the training programs implemented in the present investigation increase muscle strength. In addition, sensorimotor and vibrational training intervention aims to transfer these peripheral gains to the functional and more complex task of balance, in order to reduce the risk of falls

IG, intervention group; CG, control group; EWGSOP, European Working Group in Sarcopenia for Older People; RM, repetition maximum; TUG, timed up go; SPPB, short physical performance battery; DXA, dual energy Xray absorptiometry; AWGS, Asian working group for Sarcopenia; BIA, bioimpedance analyzer; HHD, hand held dynamometer; RPE, rating of perceived exertion; RT, resistance training; AE, adverse events; HGS, hand grip strength; GS, gait speed; BMI, body mass index; QOL, quality of life; ET, endurance training, Berg balance scale; MRI, magnetic resonance imaging

**Table 3:** Prescription components for Balance exercises (*n* = 9)

Author (s) details, Study design and settings	Participants (number and age)	Diagnostic criteria	Single/multicomponent program	Frequency/week	Intensity	Time/session	Total duration (weeks)	Type of exercise(s)	Mode of exercise(s)	Progression	Outcome measure(s)	Adverse event(s)	Educational aids/technology used	Finding(s)
Caballero-García et al. [39], Placebo controlled trial and Health centers	*N* = 44 Placebo group—22 Citrulline-malate supplemented group—22 Avg age—M 64.8 ± 3.6, F—65.4 ± 4.4	NR	Multicomponent	NR	Level of effort 3	5 min	6	Aerobic resistance Aerobic endurance Balance	Balance—standing and monopodial exercises	NR	6 min test (endurance) on 400-m track, HGS (JAMAR digital Dynamometer, Gait speed (4-m test), Squat, SPPB, Balance (Standing, semi tandem, tandem stand)	NR	NR	No significant difference in the outcome measures between placebo and intervention group
Chow et al. [22], RCT and Community dwelling	EXS + HMB: *n* = 48, CG: *n* = 48 and Vibration + HMB: *n* = 48	AWGS	Single component	3/week	35 Hz	20 min per session	12	Resistance exercise to one group and Vibration exercise to another group	Vibration platform will be used	NR	Primary outcome: Knee extension strength; Secondary outcome: HGS, GS, MM, Balancing activity, TUG test, SARC-F, SF-36, Food frequency questionnaire, activity tracker (steps)	Protocol	Wrist worn activity tracker to record daily activity	Protocol
Makizako et al. [17], RCT and Community dwelling	IG: *n* = 33(Avg age-74.1 ± 6.6, CG: *n* = 34 (Avg age-75.8 ± 7.3)	AWGS	Multicomponent	NR	NR	20–25 min of balance and aerobic	12	Resistance training, Balance, flexibility, and aerobic exercises	Tandem stand, heel-up stand, one-leg stand, weight shifts, and stepping (anterior–posterior and lateral), to improve static and dynamic balance ability	NR	Physical performance: Grip strength (HHD), Gait speed (6-m test), 5-Chair stand test, TUG and Muscle CSA and volume (MRI)	NR	Infrared timer for gait speed assessment (Outcome measure) Booklet	12 week multicomponent exercise program with progressive resistance training generally improves physical function in CDOA with sarcopenia or pre-sarcopenia. However, it is unclear whether effective in increasing muscle mass
Liang et al. [18], RCT and Post-acute care unit	IG (*n* = 30), CG (*n* = 29) Avg. Age—87.3 ± 5.4 years	AWGS	Multicomponent	2/week	NR	20 min	12	Resistance exercise and balance exercise	Balance exercise program included: heel and toe raise and static balance varied directional quick stepping, reaching and single leg standing, heel to toe walking and complex cross-over stepping activities	Week 1–3: Heel and toe raise and static balance Week 4–6: Varied directional quick stepping, Week 7–9:Reaching and single leg standing, Week 10–12:Heel to toe walking and complex cross stepping activities	Primary outcomes: Activities of daily living (Barthel index) and number of fallers; Secondary outcomes: SPPB, 4-m gait speed, HGS (Digital grip dynamometer), Berg balance, TUG, and any adverse events	NR	NR	Compared with resistance exercise, the mixed exercise program (Balance plus resistance exercise) appeared to have improved the ADL, strength, and physical performance in older sarcopenic patient in post-acute care settings
Najafi et al. [35], RCT and Nursing home	IG: *n* = 35 CG: *n* = 28 (Avg. Age—72.5 ± 7.0)	NR	Multicomponent	3/week	NR	20 min	8	Strength, walking, balance, endurance activities	Regular PA include—daily walking for 30 min plus stretching Fun PA group—strength, balance, endurance, and walking activities (in the form of rotational movement of hands with plastic balls (also k/a beach balls), catch-a-colour rockets, wands, Audubon bird and stretch bands)	NR	Balance (BBS), 6 min walk test, Muscle strength (Dynamometer)	NR	NR	Fun PA reduces sarcopenic progression through improving balance, increasing distance walked, and strengthening muscles
Hassan et al. [28], Pilot study and Nursing care facilities	EX: *n* = 18, CG: *n* = 21 (avg age: 85.9 ± 7.5 years)	EWGSOP	Multicomponent	2/week	NR	Total duration 1 h per session including resistance exercise	24	Resistance and balance training	Heel and toe raise, varied directional quick stepping, reaching, single leg standing, static balance, heel to toe walking and complex cross over stepping activities	Progression reducing hand support, Narrowing BOS, increasing speed of activity, cognitive dual task challenge	Number of falls, Qol, functional performance (SPPB), falls efficacy and cognitive wellbeing	No adverse evet	NR	Resistance and balance exercise has positive benefits for older adults residing in nursing care facilities which may transfer to reduce disability and sarcopenia transition, but more work is needed to ensure improved program uptake among residents
Maruya et al. [16], Community dwelling	IG: *n* = 26 (69.2 ± 5.6 years); CG: *n* = 14 (68.5 ± 6.2 years)	AWGS	Multicomponent	NR	NR	20–30 min per day	24	Resistance and balance training	Lower limb resistance exercises and balance exercises were used: squats, single-leg standing, and heel raises	NR	Body composition (SMI,BMI and body fat %) using BIA, Self-reported QOL (EQ-5D, GLFS-25), Physical function (HGS, duration of single leg stand, comfortable and maximum walking speed, and knee extension strength (Handheld Dynamometer)	NR	Guidebook	A 6-month home exercise program, combining walking and resistance LL exercise, was effective in improving maximum walking speed and muscle strength in individual, in more than 60 years old with pre sarcopenia and sarcopenia
Bellomo et al. [33], RCT	Gsm: 10, RT: 10, Vam: 10, CG: 10 (Avg age—70.9 ± 5.2)	Centers for disease control and prevention	Multicomponent	2/week	NR	20 min per session	12	Global sensori motor: aerobic, balance and flexibility training; Resistance training; vibratory mechanical-acoustic focal therapy	NR	NR	Maximal isometric test (Knee extension machine); gait analysis: length of half step (cm), sway area (mm^2^), ellipse surface (mm^2^) (Pedobarographic platform)	NR	NR	All the training programs implemented in the present investigation increase muscle strength. In addition, sensorimotor and vibrational training intervention aims to transfer these peripheral gains to the functional and more complex task of balance, in order to reduce the risk of falls
Kim et al. [41], RCT, Community	E + AAS: *n* = 38 (79.5 ± 2.9 years), E: *n* = 39(79.0 ± 2.9 years), AAS: *n* = 39(79.2 ± 2.8 years), HE: *n* = 39 (78.7 ± 2.8 years)	NR	Multicomponent	2/week	NR	20 min/session	12	Resistance exercise, balance, and gait training	Balance exercise-standing on one leg, multidirectional weight shifts, tandem stand, and tandem walk Gait training-Raising the toes (dorsiflexion) during the forward swing of the leg, kicking off the floor with the ball of the foot, walking with directional changes, and gait pattern variations	NR	Body composition (BIA); Functional fitness parameter (Muscle strength and walking ability)	NR	NR	Exercise and AAS together may be effective in enhancing not only muscle strength, but also combined variables of muscle mass and walking speed and of muscle mass and strength in sarcopenic women

IG, intervention group; CG, control group; EWGSOP, European Working Group in Sarcopenia for Older People; RM, repetition maximum; TUG, timed up go; SPPB, Short Physical Performance Battery; DXA, dual energy Xray absorptiometry; AWGS, Asian working group for Sarcopenia; BIA, bioimpedance analyzer; HHD, hand held dynamometer; RPE, rating of perceived exertion; RT, resistance training; AE, adverse events; HGS, hand grip strength; GS Gait speed; BMI, body mass index; QOL, quality of life; ET, endurance training, Berg balance scale; MRI, magnetic resonance imaging

**Table 4:** Prescription components for stretching exercises (*n* = 2)

Author (s) details, Study design and settings	Participants (number and age)	Diagnostic criteria	Single/multicomponent program	Frequency/week	Intensity	Time/session	Total duration (weeks)	Type of exercise(s)	Mode of exercise(s)	Progression	Outcome measure(s)	Adverse event(s)	Educational aids/technology used	Finding(s)
Makizako et al. [17], RCT and Community dwelling	IG: *n* = 33(Avg age-74.1 ± 6.6, CG: *n* = 34(Avg age-75.8 ± 7.3)	AWGS	Multicomponent	NR	NR	NR	12	Resistance training, Balance, flexibility, and aerobic exercises	NR	NR	Physical performance: Grip strength (HHD), Gait speed (6-m test), 5-Chair stand test, TUG and Muscle CSA and volume (MRI)	No AE	Infrared timer for gait speed assessment (Outcome measure) Booklet	12 week multicomponent exercise program with progressive resistance training generally improves physical function in CDOA with sarcopenia or pre-sarcopenia. However, it is unclear whether effective in increasing muscle mass
Bellomo et al. [33], RCT	Gsm: 10, RT: 10, Vam: 10, CG: 10 (Avg age-70.9 ± 5.2)	Center for disease control and prevention	Multicomponent	NR	NR	NR	12	Global sensori motor: aerobic, balance and flexibility training; Resistance training; Vibratory mechanical-acoustic focal therapy	Stretching exercises for the muscles of the lower limbs	NR	Maximal isometric test (knee extension machine); Gait analysis: Length of half step (cm), Sway area (mm^2^), Ellipse surface (mm^2^) (Pedobarographic platform)	NR	NR	All the training programs implemented in the present investigation increase muscle strength. In addition, sensorimotor and vibrational training intervention aims to transfer these peripheral gains to the functional and more complex task of balance, in order to reduce the risk of falls

IG, intervention group; CG, control group; TUG, timed up go; AWGS, Asian working group for Sarcopenia; HHD, hand held dynamometer; AE, adverse events; CSA, cross sectional area; MRI, magnetic resonance imaging

Seven studies (*n* = 7) [17, 24, 30, 33, 35, 36, 39] used aerobic and endurance exercises as a multicomponent exercise intervention program. Of the seven studies, two (*n* = 2) [30, 33] used the percentage of heart rate maximum (HR max), two (*n* = 2) [36, 39] used RPE criteria for quantifying the intensity of aerobic exercises, and the remaining three studies (*n* = 3) [17, 24, 35] did not report how the intensity was monitored. The duration of aerobic exercises ranged from 6 to 30 min per session for 2–5 days/week. Among the aerobic and endurance studies included in this review, only one study (*n* = 1) [36] reported exercise progression in terms of time/session, intensity, and rating of perceived exertion.

Balance exercises were used as a component of the multicomponent program in nine studies (*n* = 9) [16–18, 22, 28, 33, 35, 39, 41] and among those only one (*n* = 1) reported the intensity of balance exercises [39]. The level of effort was mentioned as 3 on a scale of 10, and the time duration per session ranged from 5 to 30 min for a frequency of 2/3 per week. Only two studies (*n* = 2) included in this review mentioned the progression of the balance exercise on the bases of weeks and challenging exercise [18, 28]. Two studies (*n* = 2) [17, 33] mentioned stretching exercises for sarcopenic older adults, and among those one [17] did not report the details, whereas the other [33] mentioned stretching exercises of the lower limb muscles. However, further exercise prescription components such as frequency, intensity, time, and type of stretches were not reported in both studies.

We have proposed the recommendation which the practitioner and researcher could use while prescribing exercise program for sarcopenic older adults in Table 5.

**Table 5:** Proposed exercise recommendation for sarcopenic older adults

	Frequency	Intensity	Duration (min)^a^	Type	Progression
Resistance exercise	1–5 session/week	20–80% of 1 Repetition Maximum (1-RM) or 6–14 points on Rating of Perceived Exertion (RPE) Volume of the exercise: 1–3 sets, 10–15 reps	20–75	Upper limb: biceps curl, triceps curls, double arm pull down, shoulder abduction/flexion, bench press, military press, seated row, bent row over Lower limb: knee flexion/extension, hip abduction/extension, leg press, squatting, calf raises, kick back, lunges Trunk: abdominal crunch and back extension	3 ways: 20–30% of 1RM progressing to 70–79% 1RM Body weight to thera band to ankle weights/sand bags to machine based Changing the volume of the exercise
Aerobic exercise	2–5 session/week	50–70% of HR_max_ or 7–17 point on RPE	6–30	Walking, slow running, fixed cycle ergometer, anterior/posterior/lateral repetitive stepping, stationary bicycle	3 ways: HR_max_ from 50 to 55% progressing to 65–70% RPE from 7 points progressing to 17 Varying the exercise duration keeping intensity constant/decreasing/increasing
Balance exercise	2/3 session/week	3 point on the scale of 10	5–30	Static balance: broad base standing, feet together, tandem stand, weight shifts, heel-up stand, one-leg stand Dynamic balance: heel raise, toe raise, heel/toe waling, vibration platform, stepping (anterior–posterior and lateral), quick directional change, complex cross over stepping activity, reaching	Increasing the difficulty level of exercise example from broad base to narrow base or from statics to dynamic
Stretching exercise	Static stretching exercise during warm-up and cool down				

^a^Inclusive of warm-up, cool-down and rest period between exercise

## Discussion

This rapid review intended to synthesize the details of the exercises/exercise program prescribed for the improvement of either muscle mass or muscle strength or physical performance among sarcopenic older adults residing in either long-term care setting or in the community. A total of 27 records were summarized with regard to the details of exercises such as a single-component or multicomponent exercise program, frequency/week, intensity, duration/session, total duration of the exercise program, type of exercises, progression, adverse events reported, outcome measures used, and whether technology or other educational aids were used to deliver the program. Also, the Consensus on Exercise Reporting Template (CERT) [42] was used to evaluate the completeness of exercise reporting of the studies included in this review. Most of the studies reported the details of each exercise included in the intervention to enable replication. The exercise progression details were reported well in many of the studies, with most exercise interventions being supervised. However, the way the exercise interventions were tailored were not reported in many of the studies (Supplementary material).

Given that the studies did not use uniform diagnostic criteria for sarcopenia, the prevalence varied greatly from 10 to 30% [43] depending on the classification and cutoff point chosen. The participants in the studies included in this review may be heterogenous with regard to their fitness profile and exercise capacity. Hence, the compilation of the results of these studies would be difficult and the effectiveness of the program has to be interpreted with caution. However, there are systematic reviews that give evidence about the effect of exercise on sarcopenia management. According to the findings of those systematic reviews, the best evidence exists for multimodal or mixed training programs that include resistance training, aerobic training, and balance training, with a moderate level of evidence for resistance training alone in enhancing muscle mass, muscle strength, and physical performance in sarcopenic older persons. There was a low level of evidence for flexibility or balance alone programs, as the number of studies were limited [44–48].

Eleven studies in this review used multicomponent exercise interventions for the management of sarcopenic older adults. Since there are no exercise recommendations or guidelines for the management of sarcopenia, most of the authors have followed the recommendations for physical frailty that report the beneficial effects of a multicomponent over a single-component intervention [49]. Currently, there is a lack of consensus among practitioners with regard to the dosage of resistance training that is required to get the best results in terms of muscular strength. The frequency per week of resistance training in the studies in this review ranged from a minimum of 2 days to a maximum of 5 days per week, with most of the authors preferring 3 days per week. Two reviews were conducted on the components of exercise prescription for frail older adults and on patients with knee osteoarthritis (OA), which recommended a similar frequency of resistance exercises with significant improvements in muscle strength documented with a training frequency of 2–3 days [49, 50].

Aerobic and endurance studies (*n* = 7) included in this review are part of a multicomponent exercise intervention. The frequency of aerobic exercises for sarcopenia ranged from two to five times per week. The American College of Sports Medicine physical activity recommendation for older adults states 3–5 days per week of aerobic activity, and for older adults with knee OA, the most common recommendation that exists is to perform 3–5 days per week of cardiorespiratory training [50, 51]. Even though the minimum recommended frequency is 3 days per week, a few studies of this review have used 2 days per week; however, the effect of 2 days over 3 days per week training needs to be studied. Balance exercises have been carried out in nine studies (n = 9) of this review, in which the frequency was found to be two to three times per week, which is the same in the case of older frail adults as well for older adults with knee OA [49, 50]. Also, the US Department of Health and Human Services (2008) Physical Activity Guidelines for Americans recommends three or more times per week of balance training. For stretching exercises, even though frequency was not reported, it was a part of warm-up in most of the studies. However, one of the study recommends two to three times per week of flexibility exercises for frail older adults (41).

The intensity of resistance exercises for sarcopenic older adults ranged from 20 to 79% of 1 RM or 6 to 14 RPE in the studies included in this review. For frail older adults as per the literature, the intensity varies from 40 to 80% 1RM or somewhat hard (12–14) RPE [49]. The literature suggests that 60–80% of 1RM improves the muscle mass, while more than 80% of 1RM will further improve the muscle strength at the cost of musculoskeletal injuries [51]. The intensity of aerobic exercise prescribed for sarcopenic older adults ranged from 50 to 70% of HR_max_ or 7–17 on RPE, which is in line with the recommendation of ACSM/AHA that recommends moderate- to vigorous-intensity aerobic exercise for older adults. In this review, only one study (n = 1) reported the intensity of balance exercise, which is 3 on a rating of perceived exertion and this corresponds to the recommendations for frail older adults [49].

The duration for resistance exercise ranged from a minimum of 20 min to a maximum of 75 min in the included studies. A study has mentioned that the duration for resistance exercise ranged from 10 min for frail and 20 min for pre-frail older adults [49]. The duration of aerobic exercise per session for sarcopenic older adults reported in the studies in this review ranged from 6 to 30 min per session. As per the ACSM recommendation, moderate-intensity aerobic exercise should be performed for ≥ 30 min per day or vigorous-intensity exercise for ≥ 20 min per day [14]. The duration of balance exercise in the included studies ranged from 6 to 30 min, which covers the time prescribed for frail older adults of 20 min.

The type and mode of exercise should be chosen based on the participant's level of fitness, as well as his or her interests and available resources for better compliance and for lowering the risk of injury [51, 52]. The type of resistance training delivered in the included studies is related to training the large muscle groups of the upper limb and lower limb along with the trunk. These are in line with the recommendations by other studies as well, which recommends resistance training to be directed at the large muscle groups that are important in everyday activities incorporating arms/shoulders, chest, back, hips, and legs [14, 50, 51, 53]. Aerobic and endurance exercises have been delivered as walking, stepping, and cycle ergometry. Walking is the most popular activity among senior citizens because it requires no special skills, attire, or equipment [52]. For those with musculoskeletal problem, cycling has been preferred over walking and jogging [51]. A similar type of exercise for improving cardiorespiratory fitness has been recommended for older adults with knee OA [50]. In the included studies in this review, stretching is used as part of a warm-up/cool-down in multicomponent training program, with only one study targeted specifically. Stretching of upper, trunk, and lower limb has been suggested, in line with the recommendation by ACSM as it reduces the chance of injury among older adults [53].

The progression of resistance training reported in the studies included in this review suggests starting with a fewer number of sets, higher repetition with less intensity progression, to more sets with less number of repetitions. An author has recommended that clinicians should start their clients with higher repetitions, that is, 12–15, and at a lower intensity (55% of 1RM) and eventually progress to fewer repetitions of 4–6 at a greater intensity (> 80% of 1RM) [49]. Aerobic and endurance studies included only one study (*n* = 1) [36] in this review, which reported the endurance exercise progression in terms of increasing time/session and increasing intensity as HR_max_ with higher rating of perceived exertion. Mazzeo et al. [51] recommended starting exercise with low intensity and gradually progressing to higher levels according to tolerance and preference. Only two studies (*n* = 2) included in this review have mentioned about the progression of balance exercise on the basis of weeks and challenging exercise. A similar recommendation by the US Department of Health and Human Services (2008) Physical Guidelines for Americans says progressively difficult postures gradually reduce the base of support and dynamic movements perturb the center of gravity and stress the postural muscle groups [54].

Among the included studies, *n* = 4 were study protocols and *n* = 23 were completed studies. Of these 23 studies, *n* = 15 studies did not assess for any adverse events [16, 18, 20, 21, 23, 27, 33–41], *n* = 5 studies reported no adverse events [15, 17, 24, 28, 30], and only *n* = 3 studies reported adverse events because of exercise intervention [19, 26, 31]. Muscle pain, joint pain, fatigue, vertigo, and fall were the commonly reported events.

None of the studies in this review has reported a novel or a non-traditional exercise, except one study that incorporated a BFR program (blood flow restriction) [25], which we feel is a non-conventional mode of training for improving muscle strength or muscle mass. This review did not identify a study that has adopted a tailor-made exercise program with regard to the stages of sarcopenia; hence, the authors of this review recommend designing an exercise program as per the stage of sarcopenia with appropriate progression guidelines.

In this paper, the researchers have synthesized the components of the exercises/exercise program prescribed for the improvement of muscle mass/muscle strength/physical performance among sarcopenic older adults.

## Limitations

There are a few limitations to this review. First and foremost, this review has considered only the published articles available in electronic databases. Second, it considered only full-text articles as abstracts, and proceedings were excluded.

## Future recommendations

This review has done a narrative summarization of the details of the exercise for sarcopenia. In the future a systematic review with a meta-analysis could be conducted to quantify the efficacy of the exercise program. Since there are more than two types of exercises to be compared for their effect, the authors would recommend performing a network meta-analysis. Also, studies published in non-electronic databases and on gray literature could be carried out as an update to this review.

## Significance of this review

This review has synthesized the exercise components and has come up with the exercise recommendations that would benefit the interdisciplinary team to assess, design, and appraise safe and effective exercise programs for sarcopenic older adults. The exercises identified in this review are multicomponent and multimodal in nature, giving the practitioners and researchers the freedom to choose, as per the need and available resources. Also, strategies to improve and maintain adherence to exercise-based intervention have been identified, which need to be incorporated. Moreover, this review has summarized the exercises using the CERT checklist, making the recommendations replicable and transferrable across a variety of settings (hospital, gym, home, primary care, etc.); delivery methods (group, individualized, supervised, or home based), and personnels (physiotherapist,, exercise physiologist,, trainers, or others).

## Conclusion

This review would help practitioners and researchers in selecting the frequency, intensity, duration, type, mode, and progression while prescribing exercises for sarcopenic older adults. Also, this review may assist in identifying the variations in the components of exercise prescription for sarcopenic older adults as per the targeted outcome.

## Supplementary Information

Below is the link to the electronic supplementary material.

Supplementary file1 (DOCX 201 kb)

Supplementary file2 (PDF 69 kb)
