# Supplementary material for: A review of the components of exercise prescription for sarcopenic older adults
Source: Eur Geriatr Med. 2022 Sep 2;13(6):1245–80. doi: 10.1007/s41999-022-00693-7 (PMC9722805; doi:10.1007/s41999-022-00693-7)
Supplement: Supplementary file 1 — Supplementary file1 (DOCX 201 kb) [file 41999_2022_693_MOESM1_ESM.docx]

Consensus on exercise reporting template (CERT)

| Item | Description | Laddu et al., 2021 | Seo et al., 2021 | Kuptniratsaikul et al., 2021 | Chang et al., 2021 | Osuka et al., 2021 | Chiang et al., 2021 | Caballero-Garcia et al., 2021 | Chen et al., 2021 | Moghadam et al., 2020 | Makizako et al., 202 | Liang et al., 2020 | Chow et al., 2020 | Letieri et al., 2019 | Jung et al., 2019 | Cerventes et al., 2019 | Vikberg et al., 2019 | Granic et al., 2019 | Zhu et al., 2019 | Jeon et al., 2018 | Viana t al., 2018 | Najafi et al., 2018 | Hasan et al., 2016 | Hong et al., 2016 | Maruya et al., 2016 | Bellomo et al., 2013 | Sousa et al., 2013 | Kim et al., 2012 |
| --- | --- | --- | --- | --- | --- | --- | --- | --- | --- | --- | --- | --- | --- | --- | --- | --- | --- | --- | --- | --- | --- | --- | --- | --- | --- | --- | --- | --- |
| 1 | Detailed description of type of exercise equipment | 1 | 1 | 1 | 1 | 1 | 1 | 1 | 1 | 1 | 1 | 0 | 1 | 1 | 0 | 1 | 1 | 1 | 1 | 1 | 1 | 1 | 1 | 1 | 1 | 1 | 0 | 1 |
| 2 | Detailed description of the qualification, expertise and /or training | 1 | 1 | 0 | 1 | 1 | 0 | 0 | 0 | 0 | 1 | 1 | 1 | 0 | 0 | 0 | 1 | 1 | 1 | 1 | 1 | 1 | 1 | 1 | 0 | 0 | 0 | 1 |
| 3 | Describe whether exercises are performed individually or in a group | 1 | 0 | 1 | 0 | 0 | 0 | 0 | 1 | 0 | 0 | 1 | 1 | 0 | 0 | 0 | 1 | 1 | 1 | 0 | 1 | 0 | 1 | 0 | 0 | 0 | 0 | 1 |
| 4 | Describe whether exercises are supervised or unsupervised; how they are delivered | 1 | 1 | 1 | 1 | 1 | 1 | 1 | 1 | 1 | 1 | 1 | 1 | 1 | 1 | 1 | 1 | 1 | 1 | 1 | 1 | 1 | 1 | 1 | 0 | 1 | 1 | 1 |
| 5 | Detailed description of how adherence to exercise is measured and reported | 1 | 0 | 1 | 0 | 1 | 0 | 0 | 1 | 0 | 1 | 0 | 1 | 0 | 0 | 1 | 1 | 1 | 1 | 0 | 0 | 0 | 1 | 0 | 1 | 0 | 0 | 0 |
| 6 | Detailed description of motivation strategies | 1 | 0 | 1 | 1 | 1 | 0 | 0 | 0 | 0 | 0 | 1 | 1 | 0 | 0 | 0 | 0 | 0 | 0 | 0 | 0 | 0 | 1 | 1 | 0 | 0 | 0 | 0 |
| 7a | Detailed description of the decision rule(S) for determining exercise progression | 1 | 1 | 1 | 1 | 1 | 0 | 1 | 1 | 1 | 1 | 1 | 1 | 0 | 0 | 0 | 1 | 1 | 1 | 1 | 0 | 0 | 1 | 1 | 0 | 0 | 0 | 1 |
| 7b | Detailed description of how the exercise program was progressed | 1 | 1 | 1 | 1 | 1 | 0 | 0 | 1 | 1 | 1 | 0 | 1 | 1 | 1 | 1 | 1 | 1 | 0 | 1 | 1 | 0 | 1 | 1 | 0 | 1 | 0 | 1 |
| 8 | Detailed description of each exercise to enable replication | 1 | 0 | 1 | 1 | 1 | 1 | 1 | 1 | 1 | 1 | 1 | 1 | 1 | 1 | 0 | 1 | 1 | 1 | 1 | 1 | 1 | 1 | 1 | 1 | 1 | 1 | 1 |
| 9 | Detailed description of any home program component | 0 | 1 | 1 | 1 | 1 | 0 | 0 | 0 | 1 | 1 | 0 | 0 | 0 | 0 | 0 | 0 | 1 | 1 | 0 | 0 | 0 | 1 | 1 | 1 | 1 | 0 | 0 |
| 10 | Describe whether there are any nonexercised components | 0 | 0 | 1 | 1 | 1 | 1 | 1 | 1 | 1 | 0 | 0 | 0 | 0 | 0 | 0 | 1 | 1 | 1 | 0 | 0 | 0 | 1 | 0 | 0 | 0 | 0 | 1 |
| 11 | Describe the type and number of adverse events that occur during exercise | 1 | 0 | 1 | 1 | 1 | 0 | 0 | 1 | 0 | 1 | 0 | 0 | 0 | 0 | 1 | 1 | 1 | 1 | 0 | 0 | 0 | 1 | 0 | 0 | 0 | 0 | 0 |
| 12 | Describe the setting in which exercise are performed | 1 | 0 | 1 | 1 | 1 | 0 | 0 | 0 | 0 | 0 | 1 | 1 | 0 | 0 | 0 | 0 | 1 | 0 | 1 | 0 | 1 | 1 | 0 | 0 | 0 | 0 | 1 |
| 13 | Detailed description of exercise intervention | 1 | 1 | 1 | 1 | 1 | 1 | 1 | 1 | 1 | 1 | 1 | 1 | 1 | 1 | 1 | 1 | 1 | 1 | 1 | 1 | 1 | 1 | 1 | 1 | 1 | 1 | 1 |
| 14a | Describe whether exercise are generic (one size fits all) or tailored | 1 | 0 | 1 | 0 | 1 | 0 | 0 | 1 | 0 | 1 | 0 | 0 | 0 | 0 | 0 | 0 | 0 | 0 | 0 | 0 | 0 | 0 | 0 | 0 | 0 | 0 | 0 |
| 14b | Detailed description of how exercises are tailored to the individual | 1 | 0 | 1 | 0 | 1 | 0 | 0 | 0 | 0 | 1 | 0 | 0 | 0 | 0 | 0 | 0 | 0 | 0 | 0 | 0 | 0 | 0 | 0 | 0 | 0 | 0 | 0 |
| 15 | Describe the decision rule for determining the starting level | 1 | 0 | 1 | 1 | 1 | 0 | 0 | 1 | 1 | 1 | 0 | 0 | 0 | 0 | 0 | 0 | 1 | 0 | 0 | 1 | 0 | 0 | 1 | 0 | 1 | 1 | 0 |
| 16a | Describe how adherence or fidelity is assessed/measured | 1 | 0 | 0 | 0 | 0 | 0 | 0 | 1 | 0 | 0 | 0 | 0 | 0 | 0 | 0 | 0 | 1 | 0 | 0 | 0 | 0 | 0 | 0 | 0 | 0 | 0 | 0 |
| 16b | Describe the extent to which the intervention was delivered as planned | 1 | 1 | 1 | 1 | 1 | 0 | 0 | - | 1 | 1 | 0 | - | 1 | 1 | 0 | 1 | - | 1 | 0 | 0 | 1 | 1 | 1 | 1 | 0 | 0 | 1 |
| Total score |  | 17 | 8 | 17 | 14 | 17 | 5 | 6 | 13 | 10 | 14 | 8 | 11 | 6 | 5 | 6 | 12 | 15 | 12 | 8 | 8 | 7 | 12 | 11 | 6 | 7 | 4 | 11 |
| Yes=1  No=0 | | | | | | | | | | | | | | | | | | | | | | | | | | | | |

Table 6: Proforma CERT assessment form

| Author and year: Laddu et al., 2021  Title: INERTIA: a pilot study of the impact of progressive resistance training on blood pressure control in older adult with sarcopenia  Journal: Contemporary Clinical Trials  Study location: USA | | | | | | |
| --- | --- | --- | --- | --- | --- | --- |
| Item | Description | Data extraction-details | Location (pg, URL etc) | | Yes: 1  No:0 | Reasons for rating eg, not reported or not clearly describes |
|  |  |  | Primary paper (Page, table, appendix) | Others (paper, protocol, website, URL) |  |  |
| 1 | Detailed description of the type of exercise equipment | The exercise included in progressive resistance training protocol will be performed using pressurized weight machines | Page 7 | - | 1 | - |
| 2 | Detailed description of the qualification, expertise and /or training | The PRT intervention will be performed at a University exercise gym and will be delivered by a trained research staff member with an exercise physiology background and strength and conditioning certification | Page 7 | - | 1 | - |
| 3 | Describe whether exercises are performed individually or in a group | Trainers may deliver a training session to a maximum of 2 participants at a time to promote social support (and thus, self-efficacy) and to maximize pragmatic and feasibility of study design | Page 7 | - | 1 | - |
| 4 | Describe whether exercises are supervised or unsupervised; how they are delivered | Supervised and delivered by trained research staff member | Page 7 | - | 1 | - |
| 5 | Detailed description of how adherence to exercise is measured and reported | Retention activities to improve adherence to physical activity during the intervention and surveillance period (12-weeks to one-year follow up), such as raffle tickets for session attendance or completion of data collection forms, birthday cards, and quarterly newsletters. All retention activity content will be carefully tailored to the study population and designed to ensure that materials do not contain information related to the intervention or study outcomes. | Page 8 | - | 1 | - |
| 6 | Detailed description of motivation strategies | Participants will be asked to fill out a brief exercise acceptability questionnaire at the end of each session. To reinforce resistance training behaviors, participants will be offered the opportunity to train in the research gym once a month during the surveillance period (12-weeks to one-year follow up visit). During this time, exercise trainers will be present to monitor safety and answer questions but will not provide any formal exercise instruction | Page7 | - | 1 | - |
| 7a | Detailed description of the decision rule(s) for determining exercise progression | The 1-RM test will be repeated at week 6 (of the 12-week intervention, intervention group only) and at the end of the intervention (intervention and controls). Training regimen will gradually progress by workload and by relative perceived level of exertion (RPE), via the Borg scale protocol, to monitor exercise intensity during the 12-week training program. | Page 5 | - | 1 | - |
| 7b | Detailed description of how the exercise program was progressed | The resistance training intensity and number of repetitions performed with each set will be inverse as the participant progresses. To elaborate, once overload is reached (that is, the “upper limit” of the prescribed repetition range), the trainer will gradually increase the number of repetitions (i.e., 12–15 reps), and then the training load weight (resistance) by up to 5% (e.g., 2 to 5 lb. increase in weight for arm exercises, 5 to 10 lb. increase in leg exercises). Resistance will be incremented only when a subject completes 12 reps for at least two of the three total sets at a given resistance, which will be complemented by a reduction in the number of repetitions per set. Finally, once overload of the prescribed loaded weight is achieved, then increasing the number of sets per exercise, and decreasing the rest period between sets or exercises will follow.  The ultimate goal is for each participant to progress and maintain their workload to 3 sets of 8–12 repetitions performed at 80% of the initial 1-RM (RPE of 8–10). Each participant will start the 12-week training with 1 to 2 sets/exercise, resistance at 40–50% of their 1-RM, 10–15 repetitions per set.  On week 2, participants will be instructed to perform 2 sets of 8–12 repetitions with a moderate-intensity training load of 60–65% 1-RM.  By week 3, participants will perform 3 sets of 8–12 repetitions with a moderate-to-vigorous intensity training load of 60–65% 1-RM**.**  By week 4, participants will perform 3 sets of 8 to 12 repetitions with a moderate-intensity load of 70–75% 1-RM.  By week 5, participants will perform 3 sets of 8 to 12 repetitions with a moderate-intensity load of 80% 1-RM.  Weeks 6 through 12 will be dedicated towards maintaining or working towards this optimal load of 3 sets of 8 to 12 repetitions with a moderate-intensity load of 80% 1-RM. | Page 7 | - | 1 | - |
| 8 | Detailed description of each exercise to enable replication | The PRT protocol will include a combination of seven upper- and lower-extremity exercises (chest press, seated leg press, seated latissimus pull-down, knee/leg extension, shoulder press, leg curls, and calf-raises) performed in the order described, using pressurized weight machines, where applicable. To optimize self-regulation, participants will attend exercise sessions on 2 non-consecutive days a week. | Page 7 | - | 1 | - |
| 9 | Detailed description of any home program component | Not reported | - | - | 0 | Not reported |
| 10 | Describe whether there are any nonexercised components | Not reported | - | - | 0 | Not reported |
| 11 | Describe the type and number of adverse events that occur during exercise | Participants will also be asked to report any changes in their health or adverse events occurring during one-year follow-up surveillance period (from 12-weeks to one-year follow up). At each exercise session, intervention participants will report any changes in self-reported health or adverse events. Adverse events discovered outside these planned evaluations (e.g., during intervention encounters) will be duly noted and followed up with, as needed, to assure participant safety. | Page 8 | - | 1 | - |
| 12 | Describe the setting in which exercise are performed | University exercise gym | Page 7 | - | 1 | - |
| 13 | Detailed description of exercise intervention | The exercise intervention delivered in the INERTIA study is based on the American Heart Association (AHA) and the American College of Sports Medicine (ACSM) recommendations for PRT for high risk older adults.  Each exercise session will begin with a 10-min warm-up of low-intensity aerobic exercise (treadmill walking or stationary bike) and flexibility and dynamic stretching targeting major muscle groups. Training sessions will be approximately 1 h and 30 min (including warm up and cool down). Finally, each training session will include calf-raise exercises to evaluate muscle endurance, with the goal of seeing how many calf raises a participant can do until they reach a RPE of 6–7 or a maximum of 25 calf raises per set (whichever is first). Participants will complete 2 sets using body weight only, with one hand placed on the wall for balance and may progress to holding free weights when appropriate. Each session will end with a 5-min cool-down of low-intensity static stretching, and flexibility exercises | Page 7 | - | 1 | - |
| 14a | Describe whether exercise are generic (one size fits all) or tailored | Each participant’s training program will be tailored according to their physical and functional abilities assessed at baseline, as well as personal preferences. | Page 7 | - | 1 | - |
| 14b | Detailed description of how exercises are tailored to the individual | Enrolled participants will complete a series of one-repetition maximum (1-RM) tests in a research-dedicated gym, under direct supervision of a trained exercise interventionist, and following the recommended guidelines for RM testing | Page 5 | - | 1 | - |
| 15 | Describe the decision rule for determining the starting level | One-repetition maximum (1-RM) will be assessed for the enrolled participants | Page 5 | - | 1 | - |
| 16a | Describe how adherence or fidelity is assessed/measured | All research staff carrying out recruitment will follow a detailed informed consent process that will include a careful review of the study requirements, explanation of the study protocol including the randomized assignment to the intervention or treatment, details and expectations of the treatment, and will stress importance to completing baseline, and all follow-up assessment visits regardless of whether treatment adherence is less than optimal. | Page 8 | - | 1 | - |
| 16b | Describe the extent to which the intervention was delivered as planned | Study Protocol | Page 1 | - | 1 | - |
| Total score |  |  |  |  | 17 |  |

| Author and year: Seo et al., 2021  Title: Effect of 16 weeks of resistance training on muscle quality and muscle growth factors in older adult women with sarcopenia: A RCT  Journal: International Journal of Environmental Research and Public Health  Study location: South Korea | | | | | | |
| --- | --- | --- | --- | --- | --- | --- |
| Item | Description | Data extraction-details | Location (pg, URL etc) | | Yes: 1  No:0 | Reasons for rating eg, not reported or not clearly describes |
|  |  |  | Primary paper (Page, table, appendix) | Others (paper, protocol, website, URL) |  |  |
| 1 | Detailed description of the type of exercise equipment | Training for small muscle groups was done using an elastic band (Hygenic Corporation, Akron, OH, USA) resistance exercise program | Page 5 | - | 1 | - |
| 2 | Detailed description of the qualification, expertise and /or training | The resistance training program was performed three times per week over the sixteen weeks (48 sessions) under the certified strength and conditioning specialist (NSCA-CSCS) | Page 5 | - | 1 | - |
| 3 | Describe whether exercises are performed individually or in a group | Not reported |  | - | 0 | Not Reported |
| 4 | Describe whether exercises are supervised or unsupervised; how they are delivered | The resistance training program was performed under the supervision of certified strength and conditioning specialist | Page 5 | - | 1 | - |
| 5 | Detailed description of how adherence to exercise is measured and reported | Not reported |  | - | 0 | Not Reported |
| 6 | Detailed description of motivation strategies | Not reported |  | - | 0 | Not Reported |
| 7a | Detailed description of the decision rule(s) for determining exercise progression | Intensity OMNI Scale/Color; week1-2: 4/yellow; week 3-4: 5/yellow; week 5-8: 6/yellow; week 9-12: 7/yellow; week 13-16: 8/yellow; | Page 6 | - | 1 | - |
| 7b | Detailed description of how the exercise program was progressed | The training load was increased by progressive overload and the OMNI resistance for active muscle scale (OMNI-RES AM, 0-extremely easy to 10-extremely hard) was used | Page 5 | - | 1 | - |
| 8 | Detailed description of each exercise to enable replication | Each training session  **Warm-up (5 min):**  Whole body stretching and walking  **Resistance exercise (50 min):**  **Upper body:**  Shoulder press, Front raise,  Lateral raise, Biceps curl,  Triceps extension, Kick back,  Crunch, Bent over row,  Seated row, Back extension (prone), Push up (beginner)  **Lower body:** Squat, Lunge, Lying leg abduction, Leg kick back, Pelvic lift, Leg raise,  Toe & Heel raise  **Cool-down (5 min):**  Whole body static stretching | Page 6 | - | 0 | Not Reported |
| 9 | Detailed description of any home program component | Participants were asked to maintain their usual daily activities. | Page 5 | - | 1 | - |
| 10 | Describe whether there are any nonexercised components | Not reported |  | - | 0 | Not Reported |
| 11 | Describe the type and number of adverse events that occur during exercise | Not reported |  | - | 0 | Not Reported |
| 12 | Describe the setting in which exercise are performed | Not reported |  | - | 0 | Not Reported |
| 13 | Detailed description of exercise intervention | The intervention program was conducted for 16 weeks. The resistance training program was performed three times per week over the sixteen weeks  (48 sessions). Each training session included five minutes of warm-up, fifty minutes of the resistance exercise, and five minutes of cool-down. The weight-bearing exercises described by Watanabe et al., 2015 were performed for large muscle groups and further training for small muscle groups was done using an elastic band (Hygenic Corporation, Akron, OH, USA) resistance exercise program | Page 6 | - | 1 | - |
| 14a | Describe whether exercise are generic (one size fits all) or tailored | Not reported |  | - | 0 | Not Reported |
| 14b | Detailed description of how exercises are tailored to the individual | Not reported |  | - | 0 | Not Reported |
| 15 | Describe the decision rule for determining the starting level | Not reported |  | - | 0 | Not Reported |
| 16a | Describe how adherence or fidelity is assessed/measured | Not reported |  | - | 0 | Not Reported |
| 16b | Describe the extent to which the intervention was delivered as planned | Five participants dropped out because of personal reasons or refused post-tests | Page 3 | - | 1 | - |
| Total score |  |  |  |  | 8 |  |

| Author and year: Kuptniratsaikul et al., 2021  Title: Efficacy and safety of a simple home-based resistance exercise program for older adults with low muscle mass: a prospective longitudinal clinical trial  Journal: International Journal of Rehabilitation Research  Study location: Thailand | | | | | | |
| --- | --- | --- | --- | --- | --- | --- |
| Item | Description | Data extraction-details | Location (pg, URL etc) | | Yes: 1  No:0 | Reasons for rating eg, not reported or not clearly describes |
|  |  |  | Primary paper (Page, table, appendix) | Others (paper, protocol, website, URL) |  |  |
| 1 | Detailed description of the type of exercise equipment | Body weight and Dumbbell | Page 243 | - | 1 | - |
| 2 | Detailed description of the qualification, expertise and /or training | Not described clearly | Page 242 | - | 0 | Not described clearly |
| 3 | Describe whether exercises are performed individually or in a group | Individually | Page 243 | - | 1 | - |
| 4 | Describe whether exercises are supervised or unsupervised; how they are delivered | All participants were trained in how to perform each exercise until they could perform each exercise successfully by themselves | Page 242 | - | 1 | - |
| 5 | Detailed description of how adherence to exercise is measured and reported | The number of exercise days during the 6-month study period was determined from study participant logbooks. Most participants (93%) demonstrated good compliance. Only 1.2% and 5.8% of study participants had fair and poor compliance, respectively. | Page 245 | - | 1 | - |
| 6 | Detailed description of motivation strategies | A CD and brochure were also provided to reinforce the exercise training and to serve as a reminder if needed | Page 243 | - | 1 | - |
| 7a | Detailed description of the decision rule(s) for determining exercise progression | One kilogram of weight was selected because most of our participants were slim (30–  58 kg with a mean weight of 46.8 kg), and two-thirds of them had never exercised before. We decided to choose on the side of caution to prevent exercise overload-induced muscle injury | Page 243 | - | 1 | - |
| 7b | Detailed description of how the exercise program was progressed | Patients were allowed to adjust the frequency and intensity of exercise, as tolerated | Page 243 | - | 1 | - |
| 8 | Detailed description of each exercise to enable replication | A CD and brochure were also provided to reinforce the exercise training and  to serve as a reminder if needed | Page 243 | - | 1 | - |
| 9 | Detailed description of any home program component | The simple home-based resistance exercises included in this study focused on the following muscles: intrinsic hand, around shoulder, pectorals, abdominals, back  extensor, hip flexor, hip extensor, knee extensor, ankle plantar flexor and dorsiflexor muscles | Page 242 | - | 1 | - |
| 10 | Describe whether there are any nonexercised components | To prevent cointervention, participants were asked not to perform any exercise other than the exercises prescribed by the researchers during the study period. Subjects were also asked not to receive any physiotherapy, not to take any food supplements different  from those that they normally take, or to take any medications that affect muscle mass (as previously mentioned) | Page 244 | - | 1 | - |
| 11 | Describe the type and number of adverse events that occur during exercise | The most common adverse event was muscle pain (10.3–31.5%), followed by joint pain (10.1–17.4%) and fatigue (3.4–16.3%). Other adverse event were joint swollen, cramp, dizziness and falls (unrelated to exercise) No serious adverse events were observed. | Page 245 | - | 1 | - |
| 12 | Describe the setting in which exercise are performed | Home | Page 242 | - | 1 | - |
| 13 | Detailed description of exercise intervention | Participants were asked to exercise for 30 min per session, 3–5 days/week for 24 weeks | Page 243 | - | 1 | - |
| 14a | Describe whether exercise are generic (one size fits all) or tailored | Exercises are tailored | Page 243 | - | 1 | - |
| 14b | Detailed description of how exercises are tailored to the individual | Patients were allowed to adjust the frequency and intensity of exercise, as tolerated | Page 243 | - | 1 | - |
| 15 | Describe the decision rule for determining the starting level | Participants used their body weight as resistance during the first 3 months. After that, participants began using a 1 kg dumbbell as an additional load during exercise. One kilogram of weight was selected because most of our participants were slim (30– 58 kg with a mean weight of 46.8 kg), and two-thirds of them had never exercised before. | Page 243 | - | 1 | - |
| 16a | Describe how adherence or fidelity is assessed/measured | Not reported | - | - | 0 | Not reported |
| 16b | Describe the extent to which the intervention was delivered as planned | One hundred and twelve participants met the inclusion criteria and were enrolled. Of those, 92, 89 and 85 participants were followed up at 3, 6 and 9 months, respectively | Page 242 | - | 1 | - |
| Total score |  |  |  |  | 17 |  |

| Author and year: Chang et al., 2021  Title: Effectiveness of early versus delayed exercise and nutritional intervention on segmental body composition of sarcopenic elders- A RCT  Journal: Clinical Nutrition  Study location: Taiwan | | | | | | |
| --- | --- | --- | --- | --- | --- | --- |
| Item | Description | Data extraction-details | Location (pg, URL etc) | | Yes: 1  No:0 | Reasons for rating eg, not reported or not clearly describes |
|  |  |  | Primary paper (Page, table, appendix) | Others (paper, protocol, website, URL) |  |  |
| 1 | Detailed description of the type of exercise equipment | The strengthening exercise was conducted using the pneumatic resistance training equipment (Keiser Sports Health Equipment, Fresno, CA, USA) | Page 3 | - | 1 | - |
| 2 | Detailed description of the qualification, expertise and /or training | The hospital based program was instructed and closely monitored by a group of boarded certificated physical therapists | Page 3 | - | 1 | - |
| 3 | Describe whether exercises are performed individually or in a group | Not clearly described |  | - | 0 | Not clearly described |
| 4 | Describe whether exercises are supervised or unsupervised; how they are delivered | Supervised exercise | Page 3 | - | 1 | - |
| 5 | Detailed description of how adherence to exercise is measured and reported | Not reported | - | - | 0 | Not reported |
| 6 | Detailed description of motivation strategies | During the period of home exercise, the participants were educated. The participant was provided with a digital versatile disc and a handbook containing information on  self-strengthening exercise and a balanced diet. | Page 3 | - | 1 | - |
| 7a | Detailed description of the decision rule(s) for determining exercise progression | The 1 RM was re-evaluated every week | Page 3 | - | 1 | - |
| 7b | Detailed description of how the exercise program was progressed | The 1 RM was re-evaluated every week, and the training intensity was increased progressively to a maximum of 80% 1 RM as tolerable. | Page 3 | - | 1 | - |
| 8 | Detailed description of each exercise to enable replication | The training program began with a 10-min warm-up exercise including stretching the trunk and four limbs and comfortably riding the stationary bicycle for 5 min. Later, the participants were instructed to finish 3 sets of 10 repetitions each starting from 40% of the 1 RM for leg press, leg extension, and leg curl. They were asked to rest for 2 min between each set. Each session ended with a 10-min bicycling as the cooling down exercise. | Page 3 | - | 1 | - |
| 9 | Detailed description of any home program component | Home exercise, the participants participated in a moderate-intensity exercise like walking for at least 30 min on 5 or more days a week or for a total of 150 min per week. | Page 3 | - | 1 | - |
| 10 | Describe whether there are any nonexercised components | The nutritional supplementation comprised two sticks of daily branched-chain amino acids (BCAA-Amino Vital Pro®, Ajinomoto) and two tablets daily of calcium and vitamin D3 supplement (Caltrate, Pfizer, USA) | Page 3 | - | 1 | - |
| 11 | Describe the type and number of adverse events that occur during exercise | None of the participant reported adverse event | Page 4 | - | 1 | - |
| 12 | Describe the setting in which exercise are performed | Department of physical therapy and rehabilitation in our hospital | Page 3 | - | 1 | - |
| 13 | Detailed description of exercise intervention | In the early intervention group, exercise training and nutrition supplementation were administered first followed by the home-based program. In the delayed intervention group, the sequence was reversed | Page 3 | - | 1 | - |
| 14a | Describe whether exercise are generic (one size fits all) or tailored | Not clearly described | - | - | 0 | Not clearly described |
| 14b | Detailed description of how exercises are tailored to the individual | Not clearly described | - | - | 0 | Not clearly described |
| 15 | Describe the decision rule for determining the starting level | 1 repetition maximum (RM) of leg press, leg extension, and leg curl, measured by an experienced board-certificated physical therapist. | Page 3 | - | 1 | - |
| 16a | Describe how adherence or fidelity is assessed/measured | Not reported | - | - | 0 | Not reported |
| 16b | Describe the extent to which the intervention was delivered as planned | One participant in the delayed intervention group dropped out and did not attend the first follow-up | Page 4 | - | 1 | - |
| Total score |  |  |  |  | 14 |  |

| Author and year: Osuka et al., 2021  Title: Effects of exercise and/or Beta-hydroxymethylbutyrate supplementation on muscle mass, muscle strength, and physical performance in older women with low muscle mass: A randomized double blind, placebo-controlled trial  Journal: The American Journal of clinical nutrition  Study location: Japan | | | | | | |
| --- | --- | --- | --- | --- | --- | --- |
| Item | Description | Data extraction-details | Location (pg, URL etc) | | Yes: 1  No:0 | Reasons for rating eg, not reported or not clearly describes |
|  |  |  | Primary paper (Page, table, appendix) | Others (paper, protocol, website, URL) |  |  |
| 1 | Detailed description of the type of exercise equipment | Chair based resistance training, Elastic band based resistance training, Ankle weight based resistance training and Machine based resistance training (OGWellness Technologies Co., Ltd.;Mizuno Co.) | Page 1372-1373 | - | 1 | - |
| 2 | Detailed description of the qualification, expertise and /or training | Participants allocated to the exercise programs supervised session by a certified fitness  trainer and 2 assistant trainers | Page 1372 | - | 1 | - |
| 3 | Describe whether exercises are performed individually or in a group | Not clearly described | - | - | 0 | Not clearly described |
| 4 | Describe whether exercises are supervised or unsupervised; how they are delivered | Supervised session | Page 1372 | - | 1 | - |
| 5 | Detailed description of how adherence to exercise is measured and reported | Adherence to the trial was assessed by the exercise program attendance rate and the assigned supplement daily intake | Page 1374 | - | 1 | - |
| 6 | Detailed description of motivation strategies | Participants allocated to the health education groups were provided with six 60-min sessions of health education programs that focused on preventing dementia, preventing bank transfer fraud, music therapy, appropriate nutrition and general health, oral care, and social education. All education programs were provided as lecture-style classes | Page 1373 | - | 1 | - |
| 7a | Detailed description of the decision rule(s) for determining exercise progression | A fitness trainer increased the number of sets and repetitions based on the perceived exertion of the participants. Exercise intensity was increased when the participants performed each exercise at an intensity of*<*12 points on the Borg Rate of Perceived Exertion Scale without serious fatigue or incorrect motion and posture. | Page 1373 | - | 1 | - |
| 7b | Detailed description of how the exercise program was progressed | All RTs were performed as 1–3 sets of 8–10 repetitions with gradual loading. A fitness trainer increased the number of sets and repetitions based on the perceived exertion  of the participants. The participants were asked to maintain an exercise intensity of 12–14 points on the Borg Rate of Perceived Exertion Scale | Page 1373 | - | 1 | - |
| 8 | Detailed description of each exercise to enable replication | To avoid losing the participants’ interest in an exercise routine, the RTs comprised various types of exercises, including chair-based (weeks 1–12), elastic band (weeks 5–7), ankle weight (weeks 7–12), and machine-based RT (weeks 9–12). The chair based RT included knee extension, toe raise, heel raise, knee lift, squats, lateral leg raise, and hip adduction exercises using rubber ball. Knee lift and heel raise exercises were performed progressively from a seated position to a standing position. Elastic band RT consisted of arm rowing, knee lift, and hip adduction exercises. Knee extension, heel raise, knee lift, and lateral leg exercises were provided using ankle weights of 0.5, 0.75, 1.0, or 1.5 kg based on the participant’s physical condition. In the last 4 week of the intervention, machine-based RT, including arm rowing, leg extension, hip adduction, knee extension, and trunk flexion (OGWellness Technologies Co., Ltd.;Mizuno Co.), was provided | Page 1372-1373 | - | 1 | - |
| 9 | Detailed description of any home program component | All the participants were instructed not to change their lifestyle, as much as possible, during the intervention and observation periods | Page 1372 | - | 1 | - |
| 10 | Describe whether there are any nonexercised components | All the participants were instructed to take active products including 1500 mg calcium-HMB (Ca-HMB) or placebos not including Ca-HMB produced by Kyowa Co., Ltd | Page 1373 | - | 1 | - |
| 11 | Describe the type and number of adverse events that occur during exercise | Participants recorded any exercise- or supplement-related adverse events during the intervention period using a diary. A fitness trainer also recorded adverse events during the exercise program. No exercise-related adverse events were observed. Adverse  events potentially related to the supplements included nausea, stomach discomfort, whole body discomfort, increased urinary frequency, pruritus, dizziness and high blood pressure | Page 1374 | - | 1 | - |
| 12 | Describe the setting in which exercise are performed | Participants allocated to the exercise programs were provided 60 min of supervised sessions on 2 non consecutive days per week for 12 consecutive weeks at the Tokyo Metropolitan Institute of Gerontology (TMIG) | Page 1372 | - | 1 | - |
| 13 | Detailed description of exercise intervention | Exercise sessions consisted of 5min stretching as a warm-up exercise, 50 min resistance training (RT) as the main exercise, and 5 min stretching as a cooldown exercise | Page 1372 | - | 1 | - |
| 14a | Describe whether exercise are generic (one size fits all) or tailored | Tailored | Page 1373 | - | 1 | - |
| 14b | Detailed description of how exercises are tailored to the individual | A fitness trainer and 2 assistant trainers verified the proper motion and posture of exercises and observed each participant’s fitness level. The chair-based, elastic band, and ankle weight RTs in this study were the same as those used in our previous studies | Page 1373 | - | 1 | - |
| 15 | Describe the decision rule for determining the starting level | A fitness trainer and 2 assistant trainers verified the proper motion and posture of exercises and observed each participant’s fitness level. | Page 1373 | - | 1 | - |
| 16a | Describe how adherence or fidelity is assessed/measured | Not reported | - | - | 0 | Not reported |
| 16b | Describe the extent to which the intervention was delivered as planned | A total of 156 participants underwent the baseline assessment, of which 149 (95.5%) completed the postintervention assessment at week 12, and 144 (92.3%) completed the post observation assessment at week 24 | Page 1375 | - | 1 | - |
| Total score |  |  |  |  | 17 |  |

| Author and year: Chiang et al., 2021  Title: Effects of Milk or Soy milk combined with Mild resistance exercise on the muscle mass and muscle strength in very old nursing home residents with sarcopenia  Journal: MDPI Foods  Study location: Taiwan | | | | | | |
| --- | --- | --- | --- | --- | --- | --- |
| Item | Description | Data extraction-details | Location (pg, URL etc) | | Yes: 1  No:0 | Reasons for rating eg, not reported or not clearly describes |
|  |  |  | Primary paper (Page, table, appendix) | Others (paper, protocol, website, URL) |  |  |
| 1 | Detailed description of the type of exercise equipment | Resistance training using sandbags and elastic bands | Page 2 | - | 1 | - |
| 2 | Detailed description of the qualification, expertise and /or training | Not reported | - | - | 0 | Not reported |
| 3 | Describe whether exercises are performed individually or in a group | Not reported | - | - | 0 | Not reported |
| 4 | Describe whether exercises are supervised or unsupervised; how they are delivered | Supervised | Page 2 | - | 1 | - |
| 5 | Detailed description of how adherence to exercise is measured and reported | Not reported | - | - | 0 | Not reported |
| 6 | Detailed description of motivation strategies | Not reported | - | - | 0 | Not reported |
| 7a | Detailed description of the decision rule(s) for determining exercise progression | Not reported | - | - | 0 | Not reported |
| 7b | Detailed description of how the exercise program was progressed | Not reported | - | - | 0 | Not reported |
| 8 | Detailed description of each exercise to enable replication | Mild resistance exercise training program including chair exercise, resistance exercise with sandbags and elastic bands, and balance and gait training | Page 2 | - | 1 | - |
| 9 | Detailed description of any home program component | Not reported | - | - | 0 | Not reported |
| 10 | Describe whether there are any nonexercised components | Milk and soy milk groups drank 200 mL milk or soy milk | Page 2 | - | 1 | - |
| 11 | Describe the type and number of adverse events that occur during exercise | Not reported | - | - | 0 | Not reported |
| 12 | Describe the setting in which exercise are performed | Not reported | - | - | 0 | Not reported |
| 13 | Detailed description of exercise intervention | All of the groups underwent a mild resistance exercise training program for 12 weeks (3 times/week, 30 min/time) | Page 2 | - | 1 | - |
| 14a | Describe whether exercise are generic (one size fits all) or tailored | Not reported | - | - | 0 | Not reported |
| 14b | Detailed description of how exercises are tailored to the individual | Not reported | - | - | 0 | Not reported |
| 15 | Describe the decision rule for determining the starting level | Not reported | - | - | 0 | Not reported |
| 16a | Describe how adherence or fidelity is assessed/measured | Not reported | - | - | 0 | Not reported |
| 16b | Describe the extent to which the intervention was delivered as planned | Not reported | - | - | 0 | Not reported |
| Total score |  |  |  |  | 5 |  |

| Author and year: Caballero-Garcia et al., 2021  Title: L-Citrulline supplementation and exercise in the management of sarcopenia  Journal: MDPI Nutrients  Study location: Spain | | | | | | |
| --- | --- | --- | --- | --- | --- | --- |
| Item | Description | Data extraction-details | Location (pg, URL etc) | | Yes: 1  No:0 | Reasons for rating eg, not reported or not clearly describes |
|  |  |  | Primary paper (Page, table, appendix) | Others (paper, protocol, website, URL) |  |  |
| 1 | Detailed description of the type of exercise equipment | Aerobic endurance using balls, dumbbells, elastic band and steps | Page 4 | - | 1 | - |
| 2 | Detailed description of the qualification, expertise and /or training | Not reported | - | - | 0 | Not reported |
| 3 | Describe whether exercises are performed individually or in a group | Not reported | - | - | 0 | Not reported |
| 4 | Describe whether exercises are supervised or unsupervised; how they are delivered | Supervised | Page 3 | - | 1 | - |
| 5 | Detailed description of how adherence to exercise is measured and reported | Not reported | - | - | 0 | Not reported |
| 6 | Detailed description of motivation strategies | Not reported | - | - | 0 | Not reported |
| 7a | Detailed description of the decision rule(s) for determining exercise progression | For physical activity monitoring, a record was made at each of the training sessions.  One of the members of the research team participated and collected the required information at the end of each session. In this way, we assessed the type and intensity of the exercises and the subjective sensation of each participant | Page 3-4 | - | 1 | - |
| 7b | Detailed description of how the exercise program was progressed | Not reported | - | - | 0 | Not reported |
| 8 | Detailed description of each exercise to enable replication | Physical activity protocol performed in each training session:  Warm-up (10 min): General mobility, light movements, Level of effort at 4  Balance (5 min): Standing and monopodial exercises, Level of effort at 3  Aerobic endurance (10 min): Walking, slow running, Level of effort at 7  Aerobic resistance (20 min): Overload exercises, with balls, dumbbells, elastic bands, steps, level of effort at 8 | Page 4 | - | 1 | - |
| 9 | Detailed description of any home program component | Not reported | - | - | 0 | Not reported |
| 10 | Describe whether there are any nonexercised components | Supplementation was carried out with CM (3 g/day) in capsules. The placebo was  prepared with lactose and starch in capsules of the same size, weight and colour than  CM capsules. | Page 4 | - | 1 | - |
| 11 | Describe the type and number of adverse events that occur during exercise | Not reported | - | - | 0 | Not reported |
| 12 | Describe the setting in which exercise are performed | Not reported | - | - | 0 | Not reported |
| 13 | Detailed description of exercise intervention | Physical activity protocol which include balance, aerobic resistance and endurance | Page 4 | - | 1 | - |
| 14a | Describe whether exercise are generic (one size fits all) or tailored | Not reported | - | - | 0 | Not reported |
| 14b | Detailed description of how exercises are tailored to the individual | Not reported | - | - | 0 | Not reported |
| 15 | Describe the decision rule for determining the starting level | Not reported | - | - | 0 | Not reported |
| 16a | Describe how adherence or fidelity is assessed/measured | Not reported | - | - | 0 | Not reported |
| 16b | Describe the extent to which the intervention was delivered as planned | Not reported | - | - | 0 | Not reported |
| Total score |  |  |  |  | 6 |  |

| Author and year: Chen et al., 2021  Title: Efficacy of low-load resistance training combined with blood flow restriction vs. high-load resistance training on sarcopenia among community-dwelling older Chinese people: study protocol for a 3-arm randomized controlled trial  Journal: BMC  Study location: China | | | | | | |
| --- | --- | --- | --- | --- | --- | --- |
| Item | Description | Data extraction-details | Location (pg, URL etc) | | Yes: 1  No:0 | Reasons for rating eg, not reported or not clearly describes |
|  |  |  | Primary paper (Page, table, appendix) | Others (paper, protocol, website, URL) |  |  |
| 1 | Detailed description of the type of exercise equipment | Thera-band elastic bands to deliver the resistance exercises | Page 6 | - | 1 | - |
| 2 | Detailed description of the qualification, expertise and /or training | Not reported | - | - | 0 | Not reported |
| 3 | Describe whether exercises are performed individually or in a group | Group | Page 5 | - | 1 | - |
| 4 | Describe whether exercises are supervised or unsupervised; how they are delivered | The training intervention will be carried out under the supervision of researchers and trainers | Page 8 | - | 1 | - |
| 5 | Detailed description of how adherence to exercise is measured and reported | The researchers will record the attendance of each participant in each training session. And if a participant misses a scheduled training session, the session will be completed within the training week. | Page 6 | - | 1 | - |
| 6 | Detailed description of motivation strategies | Not reported | - | - | 0 | Not reported |
| 7a | Detailed description of the decision rule(s) for determining exercise progression | Both resistance training intervention groups will receive three weekly resistance training sessions for 12 weeks with training intensities specific to their group assignment | Page 6 | - | 1 | - |
| 7b | Detailed description of how the exercise program was progressed | The resistance training protocol of LRT-BFR will consist of 3 sets of 30-15-15 repetitions of each exercise, performed at an increased intensity from 20 to 30% 1RM by different Thera-band elastic bands. Subjects will have a 20-s rest interval between  sets and a 30-s rest interval between exercises  The CRT group will perform 3 sets of 15 repetitions at 60% 1RM in the first 4 weeks, 3 sets of 12 repetitions at 65% 1RM in the second 4 weeks, and 3 sets of 10 repetitions  at 70% 1RM in the third 4 weeks. They will have a 60-s rest interval between sets. The progressive resistance is also achieved by using Thera-band elastic bands with different tensions. | Page 6 | - | 1 | - |
| 8 | Detailed description of each exercise to enable replication | Low resistance training-Blood flow restriction (LRT-BFR):  Week1-4: Repetition: 30, 15, 15, Sets: 3, Intensity: 20%, Exercises: Elbow extension, elbow flexion, leg press, knee extension, Interval between sets: 30 sec, Frequency: 3 times/week  Week 5–8: Repetition: 30, 15, 15, Sets: 3, Intensity: 25%, Exercises: Elbow extension, elbow flexion, leg press, knee extension, Interval between sets: 30 sec, Frequency: 3 times/week  Week 9-12: Repetition: 30, 15, 15, Sets: 3, Intensity: 30%, Exercises: Elbow extension, elbow flexion, leg press, knee extension, Interval between sets: 30 sec, Frequency: 3 times/week    Conventional resistance training (CRT):  Week 1-4: Repetition: 15, Sets: 3, Intensity: 60%, Interval between sets: 60 sec, Frequency: 3 times/week  Week 5-8: Repetition: 12, Sets: 3, Intensity: 65%, Interval between sets: 60 sec, Frequency: 3 times/week  Week 1-4: Repetition: 15, Sets: 3, Intensity: 70%, Interval between sets: 60 sec, Frequency: 3 times/week | Page 6 | - | 1 | - |
| 9 | Detailed description of any home program component | Not reported | - | - | 0 | Not reported |
| 10 | Describe whether there are any nonexercised components | All the participants will maintain normal dietary habits during the 12-week intervention and follow-up period. They will record all their nutritional intakes including the amount of protein they took by 3-day dietary record (two weekdays and one weekend day in a week) per week on the notebook. The weekly recording work  is assisted and supervised by community works. | Page 7 | - | 1 | - |
| 11 | Describe the type and number of adverse events that occur during exercise | The adverse events (AEs) and serious adverse events (SAEs) will be recorded for all participants during each training session | Page 8 | - | 1 | - |
| 12 | Describe the setting in which exercise are performed | Not reported | - | - | 0 | Not reported |
| 13 | Detailed description of exercise intervention | Both resistance training intervention groups will receive three weekly resistance training sessions for 12 weeks with training intensities specific to their group assignment | Page 6 | - | 1 | - |
| 14a | Describe whether exercise are generic (one size fits all) or tailored | Generic (group protocol) | Page 5 | - | 1 | - |
| 14b | Detailed description of how exercises are tailored to the individual | Not clearly described | - | - | 0 | Not clearly described |
| 15 | Describe the decision rule for determining the starting level | All subjects will complete a one-repetition maximum (1RM) strength test to assess their muscle strength and to determine the intensity for resistance training in training groups | Page 5 | - | 1 | - |
| 16a | Describe how adherence or fidelity is assessed/measured | The therapists of this study will check the file and perform the intervention according  to the grouping assignments. | Page 5 | - | 1 | - |
| 16b | Describe the extent to which the intervention was delivered as planned | Study Protocol | - | - | - | - |
| Total score |  |  |  |  | 13 |  |

| Author and year: Moghadam et al., 2020  Title: THE EFFECTS OF CONCURRENT TRAINING ORDER ON SATELLITE CELL- RELATED MARKERS, BODY COMPOSITION, MUSCULAR AND CARDIORESPIRATORY FITNESS IN OLDER MEN WITH SARCOPENIA  Journal: THE JOURNAL OF NUTRITION, HEALTH & AGING  Study location: Iran | | | | | | |
| --- | --- | --- | --- | --- | --- | --- |
| Item | Description | Data extraction-details | Location (pg, URL etc) | | Yes: 1  No:0 | Reasons for rating eg, not reported or not clearly describes |
|  |  |  | Primary paper (Page, table, appendix) | Others (paper, protocol, website, URL) |  |  |
| 1 | Detailed description of the type of exercise equipment | All exercises were performed on variable resistance machines (Technogym equipment, Italy). Endurance training using cycling on a fixed-speed cycle ergometer | Page 798 | - | 1 | - |
| 2 | Detailed description of the qualification, expertise and /or training | Not reported | - | - | 0 | Not reported |
| 3 | Describe whether exercises are performed individually or in a group | Not clearly described | - | - | 0 | Not clearly described |
| 4 | Describe whether exercises are supervised or unsupervised; how they are delivered | Supervised | - | - | 1 | - |
| 5 | Detailed description of how adherence to exercise is measured and reported | Not reported | - | - | 0 | - |
| 6 | Detailed description of motivation strategies | Not reported | - | - | 0 | Not reported |
| 7a | Detailed description of the decision rule(s) for determining exercise progression | For resistance training 1-RM was assessed. A heart rate monitor (Polar S810, Polar Electro, Kempele, Finland) was used to control and measure the exercise intensities. The periodized ET protocol was adapted from previous studies | Page 798-799 | - | 1 | - |
| 7b | Detailed description of how the exercise program was progressed | Resistance training: Week 1: 2 sets, 14-16 reps/set with 40% 1RM; Week 2: 2sets, 14-16 reps/set with 45%1RM; Week 3: 2 sets, 12-14 reps with 50% 1RM; Week 4: 2sets, 12-14 reps with 55% 1 RM; Week 5: 3sets, 10-12 reps with 60% 1RM; week 6: 3 sets, 10-12 reps with 65 % 1RM; week 7: 3sets, 8-10 reps with 70% 1RM; week 8: 3 sets, 8-10 reps with 75% 1RM [weekly frequency was 3 times/week]  Endurance training: week 1: 15 min, 55% HRmax, 11 RPE; Week 2: 15 min, 55% HRmax, 11 RPE; Week 3: 20 min, 60 HRmax, 13 RPE; Week 4: 20 min, 60% HRmax, 13 PRE; week 5: 25 min, 65% HRmax, 15 RPE; week 6: 25 min, 65% HRmax, 15 RPE; week 7: 30 min, 70% HRmax, 17 RPE; week 8: 30 min, 70% HRmax, 17 RPE [Weekly frequency was 3 times/week] | Page 798 | - | 1 | - |
| 8 | Detailed description of each exercise to enable replication | Before the beginning of the intervention, participants in the E+R and R+E groups performed 1 week of concurrent training, consisting of three exercise sessions, for familiarization purposes. This phase allowed for the instruction of correct lifting technique and familiarization with all exercises and equipment.  The specific exercises included in the program were leg extension, leg curl, bench press, lat pulldown, lateral raise, and abdominal crunch . The periodized protocols were adapted from a previous study that investigated older sarcopenic individuals and followed recommendations of the National Strength and Conditioning Association  The ET program consisted of cycling on a fixed-speed cycle ergometer. The periodized ET protocol was adapted from previous studies on older sarcopenic individuals and followed recommendations by the National Strength and Conditioning Association | Page 798 | - | 1 | - |
| 9 | Detailed description of any home program component | Participants were instructed not to alter their regular lifestyle and dietary habits throughout the study. | Page 797 | - | 1 | - |
| 10 | Describe whether there are any nonexercised components | The participants consumed a banana, providing ~0.30-0.35 g of carbohydrate per kilogram of body weight, as a pre-exercise snack ~1 hour before the training session. Additionally, dinner was consumed ~1.5-2 hours after each training session and was standardized to contain 1.7 g/kg of body weight of carbohydrate, 0.3 g/kg of body weight of protein, 0.4g /kg of body weight of fat (36). These recommendations were based on the Academy of Nutrition and Dietetics, Dietitians of Canada and the American College of Sports Medicine recommendations for macronutrient distribution (55-65% of total calories from carbohydrates, <35% of total calories from fats and 10-15% of total calories from protein). Protein timing was also considered and implemented during the study period, with ~0.25 to 0.3 g of protein/kg of body weight consumed within 2 hours after each exercise session. Participants were required to submit 3-day (2 weekdays and 1 weekend) food records at baseline and near the completion of the assigned intervention. | Page 799 | - | 1 | - |
| 11 | Describe the type and number of adverse events that occur during exercise | Not reported | - | - | 0 | Not reported |
| 12 | Describe the setting in which exercise are performed | Not reported | - | - | 0 | Not reported |
| 13 | Detailed description of exercise intervention | Participants in the Endurance (E) + Resistance (R) and R+E groups completed supervised training 3 times a week for 8 weeks, with sessions separated by at least 48 hours. The E+R group performed ET first, followed by RT; while the R+E group performed RT first, and followed by ET.  Preparatory phase followed by resistance training program and endurance training program | Page 799 | - | 1 | - |
| 14a | Describe whether exercise are generic (one size fits all) or tailored | Not clearly described | - | - | 0 | Not clearly described |
| 14b | Detailed description of how exercises are tailored to the individual | Not reported | - | - | 0 | Not reported |
| 15 | Describe the decision rule for determining the starting level | For resistance training, the participants performed two attempts and their highest lifted weight and number of repetitions were recorded. The one- repetition maximum (1RM) was estimated using a prediction formula based on the number of repetitions performed to volitional fatigue using a given weight. 1RM= weight/ (1.0278– 0.0278×reps)  For power testing, the 30-second Wingate test on a cycle ergometer (Monark, 894E) was used to measure upper and lower body maximal power | Page 799 | - | 1 | - |
| 16a | Describe how adherence or fidelity is assessed/measured | Not reported | - | - | 0 | Not reported |
| 16b | Describe the extent to which the intervention was delivered as planned | Participants in the E+R and R+E groups completed 100% of exercise sessions. | Page 799 | - | 1 | - |
| Total score |  |  |  |  | 10 |  |

| Author and year: Makizako et al., 2020  Title: Effects of a Multicomponent Exercise Program in Physical Function and Muscle Mass in Sarcopenic/Pre-Sarcopenic Adults  Journal: Journal of Clinical Medicine  Study location: Japan | | | | | | |
| --- | --- | --- | --- | --- | --- | --- |
| Item | Description | Data extraction-details | Location (pg, URL etc) | | Yes: 1  No:0 | Reasons for rating eg, not reported or not clearly describes |
|  |  |  | Primary paper (Page, table, appendix) | Others (paper, protocol, website, URL) |  |  |
| 1 | Detailed description of the type of exercise equipment | Resistance bands (TRIPLE TREE, Carbro Flavor USA Inc., CA, USA) | Page 4 | - | 1 | - |
| 2 | Detailed description of the qualification, expertise and /or training | Exercise groups were divided into two classes conducted by physiotherapists and instructors at a community center | Page 4 | - | 1 | - |
| 3 | Describe whether exercises are performed individually or in a group | Not clearly described | - | - | 0 | Not clearly described |
| 4 | Describe whether exercises are supervised or unsupervised; how they are delivered | Supervised | Page 4 | - | 1 | - |
| 5 | Detailed description of how adherence to exercise is measured and reported | Exercise class attendance rate was calculated through the 12 exercise sessions as an exercise program adherence. Among the 72 randomized participants, 67 (93.1%) completed the trial. The mean participation rate was 81% for the 12 exercise sessions. | Page 4 | - | 1 | - |
| 6 | Detailed description of motivation strategies | Not reported | - | - | 0 | Not reported |
| 7a | Detailed description of the decision rule(s) for determining exercise progression | Individuals’ strength performance was tested every two weeks to determine the resistance load (intensity of resistance bands) and accordingly increase it for the next two weeks | Page 4 | - | 1 | - |
| 7b | Detailed description of how the exercise program was progressed | Before each session, the participants checked their vital signs, including blood pressure, pulse rate, and self-reported physical condition. If vital signs were unsuitable, such as systolic blood pressure _ 180 mmHg, diastolic blood pressure _ 110 mm Hg,  or resting pulse rate _ 110 bpm or _ 50 bpm, participants were asked to avoid exercise that day. Each session began with a brief warm-up involving stretching, followed by 25 to 30 min of resistance training, 20 to 25 min of balance and aerobic exercises, and 5 min of cool-down. Resistance training used a progressive sequence based on individual strength performance, starting with no resistance load (own weight) for the first two weeks. Progressive resistance was provided by resistance bands (TRIPLE TREE, Carbro Flavor USA Inc., CA, USA) that had five resistance levels. Individuals’ strength  performance was tested every two weeks to determine the resistance load (intensity of resistance bands) and accordingly increase it for the next two weeks. In the strength performance test, participants determined their suitable resistance load at 12 to 14 on the Borg rate of perceived exertion scale, through ten repetitions of knee extensions. | Page 4 | - | 1 | - |
| 8 | Detailed description of each exercise to enable replication | For each resistance exercise, participants completed up to ten repetitions of each movement, which included: (1) knee extension (quadriceps), (2) hip flexion (knee raises) (psoas major and iliacus), (3) hip internal rotation (gluteus medius and minimus), (4) elbow flexion and shoulder abduction (trapezius and rhomboid), (5) elbow flexion and trunk rotation (pectoralis major and oblique abdominis), (6) hip extension (gluteus maximus), (7) knee flexion (hamstrings), (8) hip abduction (gluteus medius), and (9) squat (quadriceps, gluteus maximus, and hamstrings).  Balance training included a tandem stand, heel-up stand, one-leg stand, weight shifts,  and stepping (anterior-posterior and lateral), to improve static and dynamic balance ability.  Aerobic exercise consisted of anterior-posterior or lateral stepping repetitions for six minutes | Page 4 | - | 1 | - |
| 9 | Detailed description of any home program component | The participants also performed daily home-based exercises, which were self-monitored using booklets, and were encouraged to record an exercise calendar | Page 4 | - | 1 | - |
| 10 | Describe whether there are any nonexercised components | Nor reported | - | - | 0 | Not reported |
| 11 | Describe the type and number of adverse events that occur during exercise | No adverse events related to the intervention were reported | Page 6 | - | 1 | - |
| 12 | Describe the setting in which exercise are performed | Not reported | - | - | 0 | Not reported |
| 13 | Detailed description of exercise intervention | Individuals in the exercise training group participated in a progressive multicomponent exercise program over 12 weeks of supervised 60-min sessions. The intervention consisted of resistance training, balance, flexibility, and aerobic exercises. | Page 4 | - | 1 | - |
| 14a | Describe whether exercise are generic (one size fits all) or tailored | Tailored | Page 4 | - | 1 | - |
| 14b | Detailed description of how exercises are tailored to the individual | Resistance training used a progressive sequence based on individual strength performance | Page 4 | - | 1 | - |
| 15 | Describe the decision rule for determining the starting level | Resistance training used a progressive sequence based on individual strength performance | Page 4 | - | 1 | - |
| 16a | Describe how adherence or fidelity is assessed/measured | Not reported | - | - | 0 | Not reported |
| 16b | Describe the extent to which the intervention was delivered as planned | Out of 36 participant in intervention group 3 lost to follow up 1 because of physical deconditioning and 2 because of inconvinient | Page 3 | - | 1 | - |
| Total score |  |  |  |  | 14 |  |

| Author and year: Liang et al., 2020  Title: A randomized controlled trial of resistance and balance exercise for sarcopenic patients aged 80–99 years  Journal: Scientific Reports  Study location: China | | | | | | |
| --- | --- | --- | --- | --- | --- | --- |
| Item | Description | Data extraction-details | Location (pg, URL etc) | | Yes: 1  No:0 | Reasons for rating eg, not reported or not clearly describes |
|  |  |  | Primary paper (Page, table, appendix) | Others (paper, protocol, website, URL) |  |  |
| 1 | Detailed description of the type of exercise equipment | Not reported | - | - | 0 | - |
| 2 | Detailed description of the qualification, expertise and /or training | Experienced physiotherapist | Page 2 | - | 1 | - |
| 3 | Describe whether exercises are performed individually or in a group | The exercise interventions were individually designed | Page 2 | - | 1 | - |
| 4 | Describe whether exercises are supervised or unsupervised; how they are delivered | An experienced physiotherapist provided instructions and encouragement, supervising each patient session during their exercise | Page 2 | - | 1 | - |
| 5 | Detailed description of how adherence to exercise is measured and reported | Not reported | - | - | 0 | Not reported |
| 6 | Detailed description of motivation strategies | Before the start of intervention, participants in both groups and their caregivers were educated and familiarized with the training procedures. An experienced physiotherapist provided instructions and encouragement | Page 2 | - | 1 | - |
| 7a | Detailed description of the decision rule(s) for determining exercise progression | The individual loads of resistance training were determined based on the strength test at the first intervention and at the 13th session | Page 2 | - | 1 | - |
| 7b | Detailed description of how the exercise program was progressed | Not reported | - | - | 0 | Not reported |
| 8 | Detailed description of each exercise to enable replication | In the intervention group the balance exercise program included: heel and toe raise and static balance in weeks 1–3; varied directional quick stepping in weeks 4–6; reaching and single leg standing in weeks 7–9; heel to toe walking and complex cross-over stepping activities in weeks 10–12. The resistance exercise included leg press, leg extension and flexion, leg abduction and adduction, chest press, and seated row  The control group engaged in a resistance exercise program. In each session, participants received a light 5-min warm-up first followed by 20 min of resistance training. Next, they were allotted a five-minute rest before another 20 min of resistance training. Each session also ended with a 5-min cool-down. The resistance exercise was the same as the intervention group | Page 2 | - | 1 | - |
| 9 | Detailed description of any home program component | Not reported | - | - | 0 | Not reported |
| 10 | Describe whether there are any nonexercised components | Not reported | - | - | 0 | Not reported |
| 11 | Describe the type and number of adverse events that occur during exercise | Not reported | - | - | 0 | Not reported |
| 12 | Describe the setting in which exercise are performed | Post-acute care unit of the center of gerontology and geriatrics in a tertiary public hospital in Chengdu, China | Page 2 | - | 1 | - |
| 13 | Detailed description of exercise intervention | The intervention group received a mixed exercise program including balance and resistance exercise. In each session, the participants received a light 5-min warm-up followed by 20 min of targeted balance training. Next, they were allotted a five-minute rest before another 20 min of resistance training. Each session ended with a 5-min cool-down that incorporated stretching. Resistance exercise was performed at 70–80% of one-repetition maximum, 3 sets of 8–12 repetitions each (with a 2-min rest between sets) | Page 2 | - | 1 | - |
| 14a | Describe whether exercise are generic (one size fits all) or tailored | Not reported | - | - | 0 | Not reported |
| 14b | Detailed description of how exercises are tailored to the individual | Not reported | - | - | 0 | Not reported |
| 15 | Describe the decision rule for determining the starting level | Not reported | - | - | 0 | Not reported |
| 16a | Describe how adherence or fidelity is assessed/measured | Not reported | - | - | 0 | Not reported |
| 16b | Describe the extent to which the intervention was delivered as planned | Not reported | - | - | 0 | Not reported |
| Total score |  |  |  |  | 8 |  |

| Author and year: Chow et al., 2020  Title: Elastic-band resistance exercise or vibration treatment in combination with Hydroxymethylbutyrate (HMB) supplement for management of sarcopenia in older people: a study protocol for single blinded RCT in Hong Kong  Journal: BMJ  Study location: Hong Kong | | | | | | |
| --- | --- | --- | --- | --- | --- | --- |
| Item | Description | Data extraction-details | Location (pg, URL etc) | | Yes: 1  No:0 | Reasons for rating eg, not reported or not clearly describes |
|  |  |  | Primary paper (Page, table, appendix) | Others (paper, protocol, website, URL) |  |  |
| 1 | Detailed description of the type of exercise equipment | Elastic band, vibration platform (V-health, Hong Kong) | Page 4 | - | 1 | - |
| 2 | Detailed description of the qualification, expertise and /or training | Qualified coach | Page 4 | - | 1 | - |
| 3 | Describe whether exercises are performed individually or in a group | Subjects enrolled in the elastic-band exercise group will be instructed with one session of group training (at baseline) and one follow-up home visit (at 4 weeks) | Page 4 |  | 1 | - |
| 4 | Describe whether exercises are supervised or unsupervised; how they are delivered | Supervised | Page 4 | - | 1 | - |
| 5 | Detailed description of how adherence to exercise is measured and reported | Compliance of the exercise programme will be monitored by a wrist-worn  activity tracker recording motion and heart rates, supplemented with a self-reported  exercise calendar as a backup measure. | Page 4 | - | 1 | - |
| 6 | Detailed description of motivation strategies | All the three groups will receive the same level of educational talks and each subject will be given a booklet containing diet and exercise strategies. Subjects will be monitored on daily activity by a wrist-worn activity tracker | Page 4 | - | 1 | - |
| 7a | Detailed description of the decision rule(s) for determining exercise progression | Each subject’s ‘multiple repetition maximum’ | Page 4 | - | 1 | - |
| 7b | Detailed description of how the exercise program was progressed | The elastic band strengths will be progressively increased from 1.3 kg to 2.1 kg (yellow to green) of tensional force as instructed by a qualified coach in our project team based on each subject’s ‘multiple repetition maximum | Page 4 | - | 1 | - |
| 8 | Detailed description of each exercise to enable replication | Participants will be provided with Thera-Bands and an exercise leaflet. Training workshops will be conducted for subjects to ensure that correct postures are maintained in self-administered training sessions  Subjects enrolled in the elastic-band exercise group will be instructed with one session of group training (at baseline) and one follow-up home visit (at 4 weeks) including instructions on 5–10 min warm-up and cool-down routines, 30 min chair-based resistance exercises using Thera band  Subjects enrolled in the vibration treatment group will be assigned a collaborating community centre close to their home equipped with a vibration platform (V-health, Hong Kong) that delivers cyclic mechanical loading at 35 Hz and 0.3 g as previously reported. | Page 4 | - | 1 | - |
| 9 | Detailed description of any home program component | Not reported | - | - | 0 | Not reported |
| 10 | Describe whether there are any nonexercised components | Not reported | - | - | 0 | Not reported |
| 11 | Describe the type and number of adverse events that occur during exercise | Not reported | - | - | 0 | Not reported |
| 12 | Describe the setting in which exercise are performed | The study will be conducted in an academic hospital in Hong Kong | Page 4 | - | 1 | - |
| 13 | Detailed description of exercise intervention | Subjects will perform the instructed exercises three times per week, 1 hour after HMB intake, for 12 weeks  For vibration treatment: Treatment scheme is 20 min per day, at least 3 days per week for 12 weeks. | Page 4 | - | 1 | - |
| 14a | Describe whether exercise are generic (one size fits all) or tailored | Not reported | - | - | 0 | Not reported |
| 14b | Detailed description of how exercises are tailored to the individual | Not reported | - | - | 0 | Not reported |
| 15 | Describe the decision rule for determining the starting level | Not reported | - | - | 0 | Not reported |
| 16a | Describe how adherence or fidelity is assessed/measured | Not reported | - | - | 0 | Not reported |
| 16b | Describe the extent to which the intervention was delivered as planned | Study Protocol | - | - | - | - |
| Total score |  |  |  |  | 11 |  |

| Author and year: Letieri et al., 2019  Title: Effect of 16-Week Blood Flow Restriction Exercise on Functional Fitness in Sarcopenic Women: A Randomized Controlled Trial  Journal: International Journal of Morphology  Study location: Brazil | | | | | | |
| --- | --- | --- | --- | --- | --- | --- |
| Item | Description | Data extraction-details | Location (pg, URL etc) | | Yes: 1  No:0 | Reasons for rating eg, not reported or not clearly describes |
|  |  |  | Primary paper (Page, table, appendix) | Others (paper, protocol, website, URL) |  |  |
| 1 | Detailed description of the type of exercise equipment | Pneumatic cuffs used for blood flow restriction | Page 60 | - | 1 | - |
| 2 | Detailed description of the qualification, expertise and /or training | Not reported | - | - | 0 | Not reported |
| 3 | Describe whether exercises are performed individually or in a group | Not reported | - | - | 0 | Not reported |
| 4 | Describe whether exercises are supervised or unsupervised; how they are delivered | The Low intensity exercise plus Blood flow restriction (LI + BFR group) trained under supervision | Page 60 | - | 1 | - |
| 5 | Detailed description of how adherence to exercise is measured and reported | Not reported | - | - | 0 | Not reported |
| 6 | Detailed description of motivation strategies | Not reported | - | - | 0 | Not reported |
| 7a | Detailed description of the decision rule(s) for determining exercise progression | Not reported | - | - | 0 | Not reported |
| 7b | Detailed description of how the exercise program was progressed | A perceived effort scale (OMNI) was used for the exercises in BFR group for adjustment of relative exercise intensities | Page 60 | - | 1 | - |
| 8 | Detailed description of each exercise to enable replication | The LI + BFR group had a volume of 75 repetitions at 20-30 % of 1RM and 3-4 sets per exercise (30, 15, 15 and repetitions with 30" rest between sets). The pneumatic cuffs were positioned in proximal portion of the limbs. Once pneumatic cuffs were inflated, they remained inflated for all exercises, including the rest periods between sets and deflated during exercise transition (90"). The selected exercises were Leg Squat, Leg Press, Leg Extension/flexion and Stand plantar flexion. The LI + BFR group trained under supervision. All interventions performed with exercises were recorded and the participants had full follow-up during the period. | Page 60 | - | 1 | - |
| 9 | Detailed description of any home program component | Not reported | - |  | 0 | Not reported |
| 10 | Describe whether there are any nonexercised components | Not reported | - | - | 0 | Not reported |
| 11 | Describe the type and number of adverse events that occur during exercise | Not reported | - | - | 0 | Not reported |
| 12 | Describe the setting in which exercise are performed | Not reported | - | - | 0 | Not reported |
| 13 | Detailed description of exercise intervention | 16 weeks direct intervention with training frequency of 3 times per week  and 2 weeks for initial and final evaluations. Each session had a maximum duration of 40-50 minutes divided into three parts: a) 10 minutes to warm-up, with standing or sitting exercises of joint mobilization and exercises to promote respiratory body awareness; b) 20 minutes of direct intervention | Page 60 | - | 1 | - |
| 14a | Describe whether exercise are generic (one size fits all) or tailored | Not reported | - | - | 0 | Not reported |
| 14b | Detailed description of how exercises are tailored to the individual | Not reported | - | - | 0 | Not reported |
| 15 | Describe the decision rule for determining the starting level | Not reported | - | - | 0 | Not reported |
| 16a | Describe how adherence or fidelity is assessed/measured | Not reported | - | - | 0 | Not reported |
| 16b | Describe the extent to which the intervention was delivered as planned | Two persons lost to follow-up due to personal reasons | Page 61 | - | 1 | - |
| Total score |  |  |  |  | 6 |  |

| Author and year: Jung et al., 2019  Title: Circuit training improvements in Korean women with sarcopenia  Journal: Perceptual and Motor skills SAGE journal  Study location: Republic of Korea | | | | | | |
| --- | --- | --- | --- | --- | --- | --- |
| Item | Description | Data extraction-details | Location (pg, URL etc) | | Yes: 1  No:0 | Reasons for rating eg, not reported or not clearly describes |
|  |  |  | Primary paper (Page, table, appendix) | Others (paper, protocol, website, URL) |  |  |
| 1 | Detailed description of the type of exercise equipment | Not reported | - | - | 0 | Not reported |
| 2 | Detailed description of the qualification, expertise and /or training | Not reported | - | - | 0 | Not reported |
| 3 | Describe whether exercises are performed individually or in a group | Not reported | - | - | 0 | Not reported |
| 4 | Describe whether exercises are supervised or unsupervised; how they are delivered | Supervised | Page 4 | - | 1 | - |
| 5 | Detailed description of how adherence to exercise is measured and reported | Not reported | - | - | 0 | Not reported |
| 6 | Detailed description of motivation strategies | Not reported | - | - | 0 | Not reported |
| 7a | Detailed description of the decision rule(s) for determining exercise progression | Not reported | - | - | 0 | Not reported |
| 7b | Detailed description of how the exercise program was progressed | In Weeks 1-2, the training session lasted 25 minutes,  Weeks 3-8, it lasted 40 minutes  Weeks 9-12, it lasted 55 minutes  The exercise intensity levels ranged from 60% to 80% of the heart rate reserve (HRR). | Page 4 | - | 1 | - |
| 8 | Detailed description of each exercise to enable replication | The circuit training exercise consisted of 10 movements: walking in place, shoulder press and squat, twist dash, lunge, jumping jacks, kick back, push up, crunch, hip bridge, and bird dog. The program ended with a cool down period of 10 minutes. The main exercise set was performed for 10 minutes followed by five minutes of rest before the next set | Page 4 | - | 1 | - |
| 9 | Detailed description of any home program component | Not reported | - | - | 0 | Not reported |
| 10 | Describe whether there are any nonexercised components | Not reported | - | - | 0 | Not reported |
| 11 | Describe the type and number of adverse events that occur during exercise | Not reported | - | - | 0 | Not reported |
| 12 | Describe the setting in which exercise are performed | Not reported | - | - | 0 | Not reported |
| 13 | Detailed description of exercise intervention | Exercise group: Exercise intervention consisting of a warm-up period and then 25-75-minute sessions three times per week for a total of 12 weeks | Page 4 | - | 1 | - |
| 14a | Describe whether exercise are generic (one size fits all) or tailored | Not reported | - | - | 0 | Not reported |
| 14b | Detailed description of how exercises are tailored to the individual | Not reported | - | - | 0 | Not reported |
| 15 | Describe the decision rule for determining the starting level | Not reported | - | - | 0 | Not reported |
| 16a | Describe how adherence or fidelity is assessed/measured | Not reported | - | - | 0 | Not reported |
| 16b | Describe the extent to which the intervention was delivered as planned | All experimental participants (EG) completed the 12-week intervention | Page 6 | - | 1 | - |
| Total score |  |  |  |  | 5 |  |

| Author and year: Cerventes et al., 2019  Title: EFFECT OF A RESISTANCE TRAINING PROGRAM ON SARCOPENIA AND FUNCTIONALITY OF THE OLDER ADULTS LIVING IN A NURSING HOME  Journal: THE JOURNAL OF NUTRITION, HEALTH & AGING  Study location: Mexico | | | | | | |
| --- | --- | --- | --- | --- | --- | --- |
| Item | Description | Data extraction-details | Location (pg, URL etc) | | Yes: 1  No:0 | Reasons for rating eg, not reported or not clearly describes |
|  |  |  | Primary paper (Page, table, appendix) | Others (paper, protocol, website, URL) |  |  |
| 1 | Detailed description of the type of exercise equipment | Dumbbells, Resistance band | Page 830 | - | 1 | - |
| 2 | Detailed description of the qualification, expertise and /or training | Not reported | - | - | 0 | Not reported |
| 3 | Describe whether exercises are performed individually or in a group | Not reported | - | - | 0 | Not reported |
| 4 | Describe whether exercises are supervised or unsupervised; how they are delivered | Supervised | Page 830 | - | 1 | - |
| 5 | Detailed description of how adherence to exercise is measured and reported | Adherence to the exercise program was 80.55%, with seven older adults who completed 100% of the sessions. | Page 831 | - | 1 | - |
| 6 | Detailed description of motivation strategies | Not reported | - | - | 0 | Not reported |
| 7a | Detailed description of the decision rule(s) for determining exercise progression | Not reported | - | - | 0 | Not reported |
| 7b | Detailed description of how the exercise program was progressed | According to the progression of the older adult the strength and the load were increased | Page 830 | - | 1 | - |
| 8 | Detailed description of each exercise to enable replication | Not reported | - | - | 0 | Not reported |
| 9 | Detailed description of any home program component | Not reported | - | - | 0 | Not reported |
| 10 | Describe whether there are any nonexercised components | Not reported | - | - | 0 | Not reported |
| 11 | Describe the type and number of adverse events that occur during exercise | Fall | Page 833 | - | 1 | - |
| 12 | Describe the setting in which exercise are performed | Not reported | - | - | 0 | Not reported |
| 13 | Detailed description of exercise intervention | The resistance training scheme was developed based on the recommendation of the American College of Sports Medicine: three times a week for 12 weeks consisting of two to three sets at a moderate or high effort or intensity with one to three minutes of rest between sets for eight to 12 repetitions (1st and 2nd month) and 15 repetitions (3rd month) with one to three minutes of rest between sets. We used dumbbells of 0.5, 1 and 3 Kg, as well as elastic bands of three resistances (medium, strong and extra strong) | Page 830 |  | 1 | - |
| 14a | Describe whether exercise are generic (one size fits all) or tailored | Not reported | - | - | 0 | Not reported |
| 14b | Detailed description of how exercises are tailored to the individual | Not reported | - | - | 0 | Not reported |
| 15 | Describe the decision rule for determining the starting level | Not reported | - | - | 0 | Not reported |
| 16a | Describe how adherence or fidelity is assessed/measured | Not reported | - | - | 0 | Not reported |
| 16b | Describe the extent to which the intervention was delivered as planned | Not reported | - | - | 0 | Not reported |
| Total score |  |  |  |  | 6 |  |

| Author and year: Vikberg et al., 2019  Title: Effects of Resistance Training on Functional Strength and Muscle Mass in 70-Year-Old Individuals With Pre-sarcopenia: A Randomized Controlled Trial  Journal: JAMDA  Study location: Sweden | | | | | | |
| --- | --- | --- | --- | --- | --- | --- |
| Item | Description | Data extraction-details | Location (pg, URL etc) | | Yes: 1  No:0 | Reasons for rating eg, not reported or not clearly describes |
|  |  |  | Primary paper (Page, table, appendix) | Others (paper, protocol, website, URL) |  |  |
| 1 | Detailed description of the type of exercise equipment | Suspension band, backpacks filled with weights, weight vests, weight belts and resistance band | Page 29 | - | 1 | - |
| 2 | Detailed description of the qualification, expertise and /or training | Two instructor but qualification not reported | Page 29 | - | 1 |  |
| 3 | Describe whether exercises are performed individually or in a group | Group of ≤ 12 participants | Page 29 | - | 1 |  |
| 4 | Describe whether exercises are supervised or unsupervised; how they are delivered | Supervised. The instructors’ role was both to make sure the exercises were performed correctly, in a safe way, and also to monitor the maintenance of intensity | Page 29 | - | 1 |  |
| 5 | Detailed description of how adherence to exercise is measured and reported | The mean intervention attendance rate to the training sessions was 91% (range, 63%-100%) | Page 31 | - | 1 |  |
| 6 | Detailed description of motivation strategies | Not reported | - | - | 0 | Not reported |
| 7a | Detailed description of the decision rule(s) for determining exercise progression | Once a week, the instructors noted the weight each participant used in every exercise  by using a luggage scale. During the training sessions, instructors asked the participants what they scored on CR-10 scale; if they scored less than 6, more weight was added progressively. By using a protocol where the weight each participant used every week was noted, participants knew where to start the next week. | Page 29 | - | 1 |  |
| 7b | Detailed description of how the exercise program was progressed | Moderate to high RT intensity was applied using the Borg CR-10 scale, with participants’ perceived exertion scoring 6 to 7 of a maximum of 10. In 2-4 weeks: The intensity of the program increased in terms of sets and resistance, with maintenance  of CR-10 scores of 6 to 7 | Page 29 | - | 1 |  |
| 8 | Detailed description of each exercise to enable replication | Suspension bands were used as support for a majority of the exercises. Please see Supplementary material for pictures and supplementary video for a short film describing the exercises performed in the RT intervention  During the sessions, 8 exercises were performed with the aim of engaging muscle groups in the whole body, with a focus on strengthening of the lower-extremity muscles using functional exercises that are relevant for activities of daily living. Also, suspension bands were used as support for a majority of the exercises. | Page 29 | - | 1 | - |
| 9 | Detailed description of any home program component | Not reported | - | - | 0 | Not reported |
| 10 | Describe whether there are any nonexercised components | A nutritional supplement (taken once a day for 10 weeks) was also offered to participants in the intervention group, but it was not a mandatory component of the program. The 250-mL liquid supplement was milk based with added milk protein, supplying 175 kcal in the form of 19 g carbohydrates, 21 g protein, and 1.5 g fat (week 1-7 of the intervention) or 10 g carbohydrates, 30 g protein, and 1.5 g fat (week 8-10 of the intervention) (Gainomax Protein Drink, Norrmejerier, Umeå, Sweden) | Page 29 | - | 1 | - |
| 11 | Describe the type and number of adverse events that occur during exercise | A participant who previously had undergone shoulder surgery experienced shoulder pain, particularly during pushups, with pain sensations also between training sessions. Another participant experienced vertigo on a few occasions during training sessions. A third participant experienced knee pain that endured for about 1 week. Most of the participants in the training group reported delayed-onset | Page 31 | - | 1 | - |
| 12 | Describe the setting in which exercise are performed | Not reported | - | - | 0 | Not reported |
| 13 | Detailed description of exercise intervention | Persons in the intervention group were assigned to participate in a 10-week instructor-led progressive RT program consisting of 3 sessions (45 minutes each) per week with groups of 12 participants.  All training sessions started with 5 to 10 minutes of whole-body warm-up exercises. During the first week of training, no weight was used; the focus was on learning the exercises in a safe way using only participants’ body weight and suspension bands. In the first week, exercises were performed in 2 sets of 12 repetitions each, followed by 3 sets of 10 repetitions each in weeks 2 to 4  In weeks 5 to 7, participants performed 4 sets of 10 repetitions each. Up until this point, participants had been instructed that concentric and eccentric muscle contractions should last for approximately 2 seconds each  In weeks 8 to 10, the focus was on muscle power training using the same exercises, although participants were instructed to perform these exercises with considerably faster muscle contractions.  During the training sessions, 2 instructors were present and supervised the training For the exercises without weights, such as resistance band exercises, markers on the floor were used to easily know where to start and how to increase resistance. Resistance bands and weight vests, weight belts, and backpacks filled with weights or water bottles were offered | Page 29 | - | 1 | - |
| 14a | Describe whether exercise are generic (one size fits all) or tailored | Not reported | - | - | 0 | Not reported |
| 14b | Detailed description of how exercises are tailored to the individual | Not reported | - | - | 0 | Not reported |
| 15 | Describe the decision rule for determining the starting level | Not reported | - | - | 0 | Not reported |
| 16a | Describe how adherence or fidelity is assessed/measured | Not reported | - | - | 0 | Not reported |
| 16b | Describe the extent to which the intervention was delivered as planned | Four of the 36 participants randomized to the intervention group dropped out before the intervention started because of lack of time, and one person dropped out after 8 weeks of the intervention because of severe disease | Page 30 | - | 1 | - |
| Total score |  |  |  |  | 12 |  |

| Author and year: Granic et al., 2019  Title: Milk and resistance exercise intervention to improve muscle function in community-dwelling older adults at risk of sarcopenia (MIlkMAN): Protocol for a pilot study  Journal: BMJ open  Study location: England | | | | | | |
| --- | --- | --- | --- | --- | --- | --- |
| Item | Description | Data extraction-details | Location (pg, URL etc) | | Yes: 1  No:0 | Reasons for rating eg, not reported or not clearly describes |
|  |  |  | Primary paper (Page, table, appendix) | Others (paper, protocol, website, URL) |  |  |
| 1 | Detailed description of the type of exercise equipment | Cycle ergometer, treadmill | Page 5 | - | 1 | - |
| 2 | Detailed description of the qualification, expertise and /or training | Supervision of an experienced exercise physiologist | Page 5 | - | 1 | - |
| 3 | Describe whether exercises are performed individually or in a group | The sessions will be completed in groups of two to four participants | Page 5 | - | 1 | - |
| 4 | Describe whether exercises are supervised or unsupervised; how they are delivered | Supervised | Page 5 | - | 1 | - |
| 5 | Detailed description of how adherence to exercise is measured and reported | In an attempt to promote participants’ engagement with RE, each will receive a training log with diagrams and short instructions with space to record the details of  the exercise completed. Each participant must complete at least 10 sessions (out of 12) to be considered compliant with the exercise programme. Compliance with the milk and control drink intervention will be calculated as a percentage of actual consumption divided by expected consumption over the 6-week intervention. | Page 6 | - | 1 | - |
| 6 | Detailed description of motivation strategies | Not reported | - | - | 0 | Not reported |
| 7a | Detailed description of the decision rule(s) for determining exercise progression | Exercise intensity, volume, frequency and duration have been determined based on recent literature38– 40 and the American College of Sports Medicine recommendations for older adults. | Page 5 | - | 1 | - |
| 7b | Detailed description of how the exercise program was progressed | Participants’ gym attendance, sets and repetitions completed, and weight lifted will  be recorded following each RE session allowing for the calculation of measures of training load (eg, volume load (number of sets×number of repetitions×weight lifted)).  In addition to measures of external training load, resistance training intensity will be monitored using participant ratings of perceived exertion | Page 6 | - | 1 | - |
| 8 | Detailed description of each exercise to enable replication | During the first RE session, participants will be familiarised with the exercises (leg press, leg curl, seated row, chest press) as well as the equipment to be used throughout the intervention with correct technique demonstrated and extensively described.  Following the initial RE session, each remaining session will begin with a 5 min warm-up performed at progressive intensity using either a cycle ergometer or treadmill. Participants will then complete 2–4 sets of 8–12 repetitions at a workload of 70%–79% 1RM for all four of the exercises listed above. Each session will conclude with a short cooldown period of low-Intensity aerobic exercise, and (except the initial session) may be completed within 30 min. | Page 5 | - | 1 | - |
| 9 | Detailed description of any home program component | Participants will be asked to maintain their usual diet, level of physical activity and lifestyle throughout the duration of the intervention period | Page 5 | - | 1 | - |
| 10 | Describe whether there are any nonexercised components | On average, 500 mL milk contains ~20 g of protein needed to stimulate muscle protein synthesis above stimulation provided by RE. | Page 6 | - | 1 | - |
| 11 | Describe the type and number of adverse events that occur during exercise | This is a low-risk study. There is a small chance of transient muscle soreness, gastrointestinal discomfort, metabolic changes and change in appetite | Page 7 | - | 1 | - |
| 12 | Describe the setting in which exercise are performed | Community leisure centre (The Parks, North Tyneside Council, North Shields, UK) | Page 5 | - | 1 | - |
| 13 | Detailed description of exercise intervention | For each RE session a time slot of ~45–60 min in duration will be allocated, with  a minimum of 48 hours between sessions. Participants will then complete 2–4 sets of 8–12 repetitions at a workload of 70%–79% 1RM for all four of the exercises | Page 5 | - | 1 | - |
| 14a | Describe whether exercise are generic (one size fits all) or tailored | Not reported | - | - | 0 | Not reported |
| 14b | Detailed description of how exercises are tailored to the individual | Not reported | - | - | 0 | Not reported |
| 15 | Describe the decision rule for determining the starting level | Participants’ one repetition maximum (1RM) will be estimated for all four exercises | Page 5 | - | 1 | - |
| 16a | Describe how adherence or fidelity is assessed/measured | Throughout the study, the principal investigator will monitor recruitment, retention and compliance figures with the core research team. The core team will meet regularly to plan and evaluate study’s day-to-day activities. Monthly meetings will be organised with the co-investigators to update on study management and progress. | Page 7 | - | 1 | - |
| 16b | Describe the extent to which the intervention was delivered as planned | Study Protocol | - | - | - | - |
| Total score |  |  |  |  | 15 |  |

| Author and year: Zhu et al., 2019  Title: Effects of exercise and nutrition supplementation in community dwelling older Chinese people with sarcopenia : A RCT  Journal: Age and Ageing  Study location: Hong Kong | | | | | | |
| --- | --- | --- | --- | --- | --- | --- |
| Item | Description | Data extraction-details | Location (pg, URL etc) | | Yes: 1  No:0 | Reasons for rating eg, not reported or not clearly describes |
|  |  |  | Primary paper (Page, table, appendix) | Others (paper, protocol, website, URL) |  |  |
| 1 | Detailed description of the type of exercise equipment | Thera band | Page 221 | - | 1 | - |
| 2 | Detailed description of the qualification, expertise and /or training | Qualified coach | Page 221 | - | 1 | - |
| 3 | Describe whether exercises are performed individually or in a group | Two group session and one home exercise session | Page 221 | - | 1 | - |
| 4 | Describe whether exercises are supervised or unsupervised; how they are delivered | Supervised | Page 221 | - | 1 | - |
| 5 | Detailed description of how adherence to exercise is measured and reported | Mean attendance rates during the 12-week group exercise program were 95.3% in the exercise program alone group and 92.8% in the combined-exercise program and nutrition supplement group. | Page 223 | - | 1 | - |
| 6 | Detailed description of motivation strategies | Not reported | - | - | 0 | Not reported |
| 7a | Detailed description of the decision rule(s) for determining exercise progression | The exercise intensity was closely monitored and adjusted by the qualified coach. | Page 221 | - | 1 | - |
| 7b | Detailed description of how the exercise program was progressed | Not reported | - | - | 0 | Not reported |
| 8 | Detailed description of each exercise to enable replication | Exercise program alone group  Two group exercise sessions and one-home exercise session were conducted on weekly basis for 12 weeks. Group exercises included 5–10 min warm-up and cool-down routine, 20–30 min chair-based resistance exercises using Thera- Bands, and 20-min aerobic exercises  Combined-exercise program and nutrition supplement group  This group received nutrition supplement and the above exercise program | Page 221 | - | 1 | - |
| 9 | Detailed description of any home program component | Participants were also provided with Thera-Bands and an exercise leaflet and were asked to keep the home exercise session between 12 to 24 weeks | Page 221 | - | 1 | - |
| 10 | Describe whether there are any nonexercised components | The nutrition supplement consisted of two sachets of Ensure NutriVigor daily from baseline to 12 weeks. Each sachet (54.1 g powder) contains 231 calories, 8.61 g protein, 1.21 g β-hydroxy β-methylbutyrate, 130 IU vitamin D and 0.29 g omega-3 fatty acid. | Page 222 | - | 1 | - |
| 11 | Describe the type and number of adverse events that occur during exercise | During the study period, four adverse events and 12 serious adverse events were reported but none were related to the prescribed intervention. | Page 222 | - | 1 | - |
| 12 | Describe the setting in which exercise are performed | Not reported | - | - | 0 | Not reported |
| 13 | Detailed description of exercise intervention | The exercise program consisted of 90-min group training twice weekly and one-home session weekly for 12 weeks. Participants in the combined group were additionally asked to consume nutrition supplement twice daily for 12 weeks. Both groups were encouraged to keep home exercise after intervention period for another 12 weeks to  detect sustained effect | Page 220 | - | 1 | - |
| 14a | Describe whether exercise are generic (one size fits all) or tailored | Not reported | - | - | 0 | Not reported |
| 14b | Detailed description of how exercises are tailored to the individual | Not reported | - | - | 0 | Not reported |
| 15 | Describe the decision rule for determining the starting level | Not reported | - | - | 0 | Not reported |
| 16a | Describe how adherence or fidelity is assessed/measured | Not reported | - | - | 0 | Not reported |
| 16b | Describe the extent to which the intervention was delivered as planned | Due to limited resources, the trial was ended before reaching the anticipated sample size. Twenty-five participants did not attend the 12-week assessment due to various reasons. Additional 12 participants were lost follow-up at 24-week assessment | Page 222 | - | 1 | - |
| Total score |  |  |  |  | 12 |  |

| Author and year: Jeon et al., 2018  Title: Effect of Squat Exercises on Lung Function in Elderly Women with Sarcopenia  Journal: Journal of Clinical Medicine  Study location: South Korea | | | | | | |
| --- | --- | --- | --- | --- | --- | --- |
| Item | Description | Data extraction-details | Location (pg, URL etc) | | Yes: 1  No:0 | Reasons for rating eg, not reported or not clearly describes |
|  |  |  | Primary paper (Page, table, appendix) | Others (paper, protocol, website, URL) |  |  |
| 1 | Detailed description of the type of exercise equipment | Mechanically-assisted squat exercise (AMF-101, Foretek Microsystem, Seoul, Korea) | Page 2 | - | 1 | - |
| 2 | Detailed description of the qualification, expertise and /or training | Experienced trainer | Page 4 | - | 1 | - |
| 3 | Describe whether exercises are performed individually or in a group | Not reported | - | - | 0 | Not reported |
| 4 | Describe whether exercises are supervised or unsupervised; how they are delivered | Supervised | Page 4 | - | 1 | - |
| 5 | Detailed description of how adherence to exercise is measured and reported | Not reported | - | - | 0 | Not reported |
| 6 | Detailed description of motivation strategies | Not reported | - | - | 0 | Not reported |
| 7a | Detailed description of the decision rule(s) for determining exercise progression | The participants were asked to score their exercise intensity during each session using the Borg scale | Page 4 | - | 1 | - |
| 7b | Detailed description of how the exercise program was progressed | Each participant was asked to assess RPE on the squat machine for 3–4 min to determine his or her squat position for the main trial. The squat position was defined as feeling between somewhat difficult and difficult (i.e., RPE of 12–16 while in the squat position). Subsequently, each participant changed position from sitting to supine to tilt, with 1–2 min rest periods. They performed 6–7 rotations of these positions, and the exercise intensity was increased slightly (i.e., the squat position time was delayed). All exercise sessions were supervised by an experienced trainer | Page 4 | - | 1 | - |
| 8 | Detailed description of each exercise to enable replication | The mechanically-assisted squat device program was as follows: Sit down—supine—tilt—squat. The program was a recursive exercise. All participants bent the hips and knees as much as possible while straightening the back (the squat position). When participants felt fatigue or discomfort in the legs, they could lean on the machine or change their position from squat to tilt or sit, aided by a mechanical device. Participants performed the exercise program for 30 min at a rating of perceived exertion (RPE) of 12–14 during weeks 1–3. After week 3, the emphasis was placed on reaching and maintaining exercise intensity at RPE of approximately 14–16 for 30 min | Page 4 | - | 1 | - |
| 9 | Detailed description of any home program component | Not reported | - | - | 0 | Not reported |
| 10 | Describe whether there are any nonexercised components | Not reported | - | - | 0 | Not reported |
| 11 | Describe the type and number of adverse events that occur during exercise | Not reported | - | - | 0 | Not reported |
| 12 | Describe the setting in which exercise are performed | Hospital | Page 4 | - | 1 | - |
| 13 | Detailed description of exercise intervention | A structured program of squat exercises was conducted for 30 mins, three times per week on alternate days, for 6 weeks at the hospital. | Page 4 | - | 1 | - |
| 14a | Describe whether exercise are generic (one size fits all) or tailored | Not reported | - | - | 0 | Not reported |
| 14b | Detailed description of how exercises are tailored to the individual | Not reported | - | - | 0 | Not reported |
| 15 | Describe the decision rule for determining the starting level | Not reported | - | - | 0 | Not reported |
| 16a | Describe how adherence or fidelity is assessed/measured | Not reported | - | - | 0 | Not reported |
| 16b | Describe the extent to which the intervention was delivered as planned | Not reported | - | - | 0 | Not reported |
| Total score |  |  |  |  | 8 |  |

| Author and year: Viana et al., 2018  Title: Effect of a resistance exercise program for sarcopenic elderly women: quasi-experimental study  Journal: Fisioterapia em Movimento  Study location: Brazil | | | | | | |
| --- | --- | --- | --- | --- | --- | --- |
| Item | Description | Data extraction-details | Location (pg, URL etc) | | Yes: 1  No:0 | Reasons for rating eg, not reported or not clearly describes |
|  |  |  | Primary paper (Page, table, appendix) | Others (paper, protocol, website, URL) |  |  |
| 1 | Detailed description of the type of exercise equipment | Ankle weight | Page 4 | - | 1 | - |
| 2 | Detailed description of the qualification, expertise and /or training | Trained physical therapist | Page 4 | - | 1 | - |
| 3 | Describe whether exercises are performed individually or in a group | Small groups of four or five elder | Page 4 | - | 1 | - |
| 4 | Describe whether exercises are supervised or unsupervised; how they are delivered | Supervised | Page 4 | - | 1 | - |
| 5 | Detailed description of how adherence to exercise is measured and reported | Not reported | - | - | 0 | Not reported |
| 6 | Detailed description of motivation strategies | Not reported | - | - | 0 | Not reported |
| 7a | Detailed description of the decision rule(s) for determining exercise progression | Not reported | - | - | 0 | Not reported |
| 7b | Detailed description of how the exercise program was progressed | Load were reassessed every two weeks according to a previously published protocol | Page 4 | - | 1 | - |
| 8 | Detailed description of each exercise to enable replication | The progressive resistance exercise program (PREP) was conducted over a period of 12 weeks, three sessions/ week and duration of an hour  The sessions were divided into three stages:  First, ten minutes of lower limbs stretching exercises (hips and knees flexors and extensors, triceps surae — three times of 30 seconds each side)  Second, 40 minutes of strengthening exercises using knee extension and flexion, hip extension and flexion, hip abduction and bridge hip plus hip abduction using a ball and semi-squat. Ankle weights were used to perform the exercises with one minute interval between the three sets of 12 repetitions each  The last stage consisted of 10 minutes cool down exercises. | Page 4 | - | 1 | - |
| 9 | Detailed description of any home program component | Not reported | - | - | 0 | Not reported |
| 10 | Describe whether there are any nonexercised components | Not reported | - | - | 0 | Not reported |
| 11 | Describe the type and number of adverse events that occur during exercise | Not reported | - | - | 0 | Not reported |
| 12 | Describe the setting in which exercise are performed | Not reported | - | - | 0 | Not reported |
| 13 | Detailed description of exercise intervention | Progressive resistance exercise program (PREP) based on 75% of the participant’s maximum load (12/week, 3 times/week). | Page 1 | - | 1 | - |
| 14a | Describe whether exercise are generic (one size fits all) or tailored | Not reported | - | - | 0 | Not reported |
| 14b | Detailed description of how exercises are tailored to the individual | Not reported | - | - | 0 | Not reported |
| 15 | Describe the decision rule for determining the starting level | For knees flexors and extensors, exercises were performed using 75% of the subject’s maximal load | Page 4 | - | 1 | - |
| 16a | Describe how adherence or fidelity is assessed/measured | Not reported | - | - | 0 | Not reported |
| 16b | Describe the extent to which the intervention was delivered as planned | Not reported | - | - | 0 | Not reported |
| Total score |  |  |  |  | 8 |  |

| Author and year: Najafi et al., 2018  Title: The Effect of Fun Physical Activities on Sarcopenia Progression among Elderly Residents in Nursing Homes: a Randomized Controlled Trial  Journal: Journal of Caring Sciences  Study location: Iran | | | | | | |
| --- | --- | --- | --- | --- | --- | --- |
| Item | Description | Data extraction-details | Location (pg, URL etc) | | Yes: 1  No:0 | Reasons for rating eg, not reported or not clearly describes |
|  |  |  | Primary paper (Page, table, appendix) | Others (paper, protocol, website, URL) |  |  |
| 1 | Detailed description of the type of exercise equipment | Plastic balls (also known as Beach Balls), Catch-a-Color Rockets, Wands, Audubon Bird, and stretch bands. | Page 138 | - | 1 | - |
| 2 | Detailed description of the qualification, expertise and /or training | A certified clinical exercise specialist and a general practitioner were present during the exercise sessions | Page 138 | - | 1 | - |
| 3 | Describe whether exercises are performed individually or in a group | Not reported |  | - | 0 | Not reported |
| 4 | Describe whether exercises are supervised or unsupervised; how they are delivered | Supervised | Page 138 | - | 1 | - |
| 5 | Detailed description of how adherence to exercise is measured and reported | Not reported | - | - | 0 | Not reported |
| 6 | Detailed description of motivation strategies | Not reported | - | - | 0 | Not reported |
| 7a | Detailed description of the decision rule(s) for determining exercise progression | Not reported | - | - | 0 | Not reported |
| 7b | Detailed description of how the exercise program was progressed | Not reported | - | - | 0 | Not reported |
| 8 | Detailed description of each exercise to enable replication | The Intervention group engaged in fun physical activity and the control group in regular physical activity for 20 minutes per session, 3 times a week for 8 weeks in the hall of the nursing homes  For the regular physical activity group, the exercises consisted of the routine activities of the nursing home such as daily walking for half an hour around the area of the nursing home and stretching.  The protocol for the fun physical activity group included strength, balance, endurance, and walking activities in the form of rotational movements of the hands with plastic balls (also known as Beach Balls), Catch-a-Color Rockets, Wands, Audubon Bird, and stretch bands. | Page 138 | - | 1 | - |
| 9 | Detailed description of any home program component | Not reported | - | - | 0 | Not reported |
| 10 | Describe whether there are any nonexercised components | Not reported | - | - | 0 | Not reported |
| 11 | Describe the type and number of adverse events that occur during exercise | Not reported | - | - | 0 | Not reported |
| 12 | Describe the setting in which exercise are performed | Hall of nursing home | Page 138 | - | 1 | - |
| 13 | Detailed description of exercise intervention | The intervention was performed for 20 minutes per session, 3 times a week for 8 weeks in the hall of the nursing homes | Page 138 | - | 1 | - |
| 14a | Describe whether exercise are generic (one size fits all) or tailored | Not reported | - | - | 0 | Not reported |
| 14b | Detailed description of how exercises are tailored to the individual | Not reported | - | - | 0 | Not reported |
| 15 | Describe the decision rule for determining the starting level | Not reported | - | - | 0 | Not reported |
| 16a | Describe how adherence or fidelity is assessed/measured | Not reported | - | - | 0 | Not reported |
| 16b | Describe the extent to which the intervention was delivered as planned | Four older adults were excluded from the control group for being absent in more than two exercise sessions, two people for disinterest in participating and one older adult because of hospitalization | Page 138 | - | 1 |  |
| Total score |  |  |  |  | 7 |  |

| Author and year: Hasan et al., 2016  Title: Impact of resistance training on sarcopenia in nursing care facilities: A pilot study  Journal: Geriatric Nursing  Study location: Australia | | | | | | |
| --- | --- | --- | --- | --- | --- | --- |
| Item | Description | Data extraction-details | Location (pg, URL etc) | | Yes: 1  No:0 | Reasons for rating eg, not reported or not clearly describes |
|  |  |  | Primary paper (Page, table, appendix) | Others (paper, protocol, website, URL) |  |  |
| 1 | Detailed description of the type of exercise equipment | Resistance training was by air-pneumatic equipment (HUR Health and Fitness Equipment, Australia) specifically designed for rehabilitation and commonly used in very old adults with disability and care needs | Page 2 | - | 1 | - |
| 2 | Detailed description of the qualification, expertise and /or training | Trained allied heal professional | Page 3 | - | 1 | - |
| 3 | Describe whether exercises are performed individually or in a group | Exercise was conducted in groups of up to 10 individuals. | Page 3 | - | 1 | - |
| 4 | Describe whether exercises are supervised or unsupervised; how they are delivered | Supervised by the trained professional who monitored group safety | Page 3 | - | 1 | - |
| 5 | Detailed description of how adherence to exercise is measured and reported | To promote project uptake and adherence, all facilities staff participating in bringing residents to and from trainings, and/or assessments, undertook project training seminars, and exercise and assessment sessions are grounded in evidence via lessons learnt by our group from previous nursing care deliveries. EX participation was tracked across the course of the intervention as session adherence. In relation to adherence, 52.4% of participants (n = 11) completed between 10% and 50% of the training protocol, six of whom did 25% or less, and only 28.4% of the group completed more than 75% of the protocol | Page 2-3 | - | 1 | - |
| 6 | Detailed description of motivation strategies | Verbal motivation | Page 3 | - | 1 | - |
| 7a | Detailed description of the decision rule(s) for determining exercise progression | Following a reduced sets and repetitions two week conditioning period, participants were prescribed 2-3 sets per exercise at a resistance they could complete 10-15 times22 with a perceived rate of exertion of 12-14 on the Borg Scale | Page 2 | - | 1 | - |
| 7b | Detailed description of how the exercise program was progressed | Exercise intensity was progressed for the resistance training exercises by increasing the load when participants could comfortably complete 3 sets of 10 repetitions or by increasing repetitions with the same load to 3 sets of 15 repetitions  For the balance exercises, progressions involved reducing hand support, narrowing the base of support, increasing the speed of the activity and/or introducing a cognitive dual-task challenge | Page 2-3 | - | 1 | - |
| 8 | Detailed description of each exercise to enable replication | **Lower- and upper-body, and the trunk exercises included:** elbow and shoulder extension (dip), leg press, knee extension and flexion, hip abduction and adduction,  abdominal curl and back extension  **Balance exercises included:** heel and toe raises, varied directional quick stepping, reaching, single leg standing, static balance, heel to toe walking and complex cross over stepping activities  Sessions lasted for 1 hour | Page 2-3 | - | 1 | - |
| 9 | Detailed description of any home program component | Not reported | - | - | 0 | Not reported |
| 10 | Describe whether there are any nonexercised components | Not reported | - | - | 0 | Not reported |
| 11 | Describe the type and number of adverse events that occur during exercise | Among the exercise group, training was associated with no adverse events | Page 4 | - | 1 | - |
| 12 | Describe the setting in which exercise are performed | Not reported | - | - | 0 | Not reported |
| 13 | Detailed description of exercise intervention | The exercise (EX) facilities were provided with twice weekly progressive resistance and balance training up to 50 h over a six month period | Page 2 | - | 1 | - |
| 14a | Describe whether exercise are generic (one size fits all) or tailored | Not reported | - | - | 0 | Not reported |
| 14b | Detailed description of how exercises are tailored to the individual | Not reported | - | - | 0 | Not reported |
| 15 | Describe the decision rule for determining the starting level | Not reported | - | - | 0 | Not reported |
| 16a | Describe how adherence or fidelity is assessed/measured | Not reported | - | - | 0 | Not reported |
| 16b | Describe the extent to which the intervention was delivered as planned | Seven individuals from the parent study EX group attended 0 exercise session and were re-allocated to the CON for the analysis of this sub-study, bringing group numbers to even (EX - 28 in the parent study to 21 in the sub-study; CON - 17 in the  parent study plus seven allocated to CON minus three pacemakers = 21 in the sub-study). Five individuals who did not attend or assent to the follow-up assessment had their data carried forward from baseline for analysis (EX, N = 2) and one individual died (EX) | Page 3 | - | 1 | - |
| Total score |  |  |  |  | 12 |  |

| Author and year: Hong et al., 2016  Title: Effects of home-based tele-exercise on sarcopenia among community-dwelling elderly adults: body composition and functional fitness  Journal: Experimental Gerontology  Study location: Republic of Korea | | | | | | |
| --- | --- | --- | --- | --- | --- | --- |
| Item | Description | Data extraction-details | Location (pg, URL etc) | | Yes: 1  No:0 | Reasons for rating eg, not reported or not clearly describes |
|  |  |  | Primary paper (Page, table, appendix) | Others (paper, protocol, website, URL) |  |  |
| 1 | Detailed description of the type of exercise equipment | Dumbbell | Page 8 | - | 1 | - |
| 2 | Detailed description of the qualification, expertise and /or training | Instructor | Page 2 | - | 1 | - |
| 3 | Describe whether exercises are performed individually or in a group | Not reported | - | - | 0 | Not reported |
| 4 | Describe whether exercises are supervised or unsupervised; how they are delivered | The supervised, progressive exercise protocol for the Tele exercise group (TEG) | Page 10 | - | 1 | - |
| 5 | Detailed description of how adherence to exercise is measured and reported | Not reported | - | - | 0 | Not reported |
| 6 | Detailed description of motivation strategies | During the intervention period, nutrition and exercise education was provided for all the participants once every four weeks. During the education session, we told all the participants to inform the instructor when any change occurred in their physical activity levels and nutrition intake | Page 9 | - | 1 | - |
| 7a | Detailed description of the decision rule(s) for determining exercise progression | Since the elderly participants had difficulty conducting a one-repetition maximum test (1RM) for the resistance training, exercise intensity was controlled using the Borg Rating of Perceived Exertion (RPE) Scale, and a combination of somewhat hard (RPE 13-14) and hard (RPE 15-16) intensity exercise was used according to the ACSM’s guidelines | Page 9 | - | 1 | - |
| 7b | Detailed description of how the exercise program was progressed | Exercise intensity was progressively increased by about 2 steps every four weeks, from RPE 11 to RPE 15 | Page 9 | - | 1 | - |
| 8 | Detailed description of each exercise to enable replication | The warm-up and cool-down included stretching and walking in place (RPE 9-11). The main exercise consisted of resistance training including bicep curls, triceps curls, front raises, leg raises, leg curls, leg extensions, squats, and calf raises, with no extra weight for Weeks 1-4, with a 1-kg dumbbell for Weeks 5-8, and with a 2-kg dumbbell for Weeks 9-12.  At that time, an all-in-one PC with Internet connection was provided and installed for the exercise participants and the instructor. Participants were also provided with folding chairs, exercise mats, dumbbells (1- kg and 2-kg), and PC operation manuals to facilitate their participation in the tele-exercise program. A post-test was conducted during the last week of the 12-week program.  These exercises targeted the major muscle groups, such as the legs, calves, back, abdomen, chest, shoulders, and arms over three sets of 8-10 repetitions. The interval between each set was less than a minute. The total exercise time was progressively increased by 20 to 40 minutes during the intervention period  The same instructor provided one-on-one instructions to each participant with the target RPE per session. Each participant turned on the PC, sat on the chair, and followed the instructor’s movements in the same body plane he or she visualized on the touchscreen during the exercise session. Triceps curls and squats were performed in the frontal plane, while calf raises, bicep curls, front raises, leg raises, leg curls, and leg extensions were performed in the sagittal plane | Page 8 | - | 1 | - |
| 9 | Detailed description of any home program component | They were encouraged throughout the intervention period to maintain the same physical activity levels and calorie intake as before participating. | Page 9 | - | 1 | - |
| 10 | Describe whether there are any nonexercised components | Not reported | - | - | 0 | Not reported |
| 11 | Describe the type and number of adverse events that occur during exercise | Not reported | - | - | 0 | Not reported |
| 12 | Describe the setting in which exercise are performed | Not reported | - | - | 0 | Not reported |
| 13 | Detailed description of exercise intervention | The tele-exercise group (TEG) performed supervised resistance exercise at home for 20–40 minutes a day three times per week for 12 weeks. The supervised, progressive exercise protocol for the TEG was designed based on the guidelines of the American College of Sports Medicine (ACSM) for resistance training with older participants. The TEG had three sessions of video conferencing-based exercise per week on non-consecutive days (separated by at least 48 hours) for 12 weeks. Each session consisted of a warm-up (5 min), a main exercise (10-30 min), and a cool-down (5 min) | Page 9 | - | 1 | - |
| 14a | Describe whether exercise are generic (one size fits all) or tailored | Not reported | - | - | 0 | Not reported |
| 14b | Detailed description of how exercises are tailored to the individual | Not reported | - | - | 0 | Not reported |
| 15 | Describe the decision rule for determining the starting level | Rating of perceived exertion (RPE) | Page 9 | - | 1 | - |
| 16a | Describe how adherence or fidelity is assessed/measured | Not reported | - | - | 0 | Not reported |
| 16b | Describe the extent to which the intervention was delivered as planned | Two participants in the tele-exercise group TEG (one due to back surgery and the other due to a no-show for the post-test) and one in the control group CTG (due to an accidental fall) dropped out | Page 8 | - | 1 | - |
| Total score |  |  |  |  | 10 |  |

| Author and year: Maruya et al., 2016  Title: Effect of a simple and adherent home exercise program on the physical function of community dwelling adults sixty years of age and older with pre-sarcopenia or sarcopenia  Journal: The Journal of Physical Therapy Science  Study location: Japan | | | | | | |
| --- | --- | --- | --- | --- | --- | --- |
| Item | Description | Data extraction-details | Location (pg, URL etc) | | Yes: 1  No:0 | Reasons for rating eg, not reported or not clearly describes |
|  |  |  | Primary paper (Page, table, appendix) | Others (paper, protocol, website, URL) |  |  |
| 1 | Detailed description of the type of exercise equipment | All components of the program were ‘easy to perform’ and did not require any special facility or equipment | Page 3187 | - | 1 | - |
| 2 | Detailed description of the qualification, expertise and /or training | Physiotherapist | - | - | 0 | Not reported |
| 3 | Describe whether exercises are performed individually or in a group | Not reported | - | - | 0 | Not reported |
| 4 | Describe whether exercises are supervised or unsupervised; how they are delivered | Not reported | - | - | 0 | Not reported |
| 5 | Detailed description of how adherence to exercise is measured and reported | Reviewing participants’ daily training calendars assessed adherence to the exercise program for participants in the intervention group. Adherence to the program of exercise was high, with completion of 70–90% of the total training volume | Page 3187 | - | 1 | - |
| 6 | Detailed description of motivation strategies | Not reported | - | - | 0 | Not reported |
| 7a | Detailed description of the decision rule(s) for determining exercise progression | Not reported | - | - | 0 | Not reported |
| 7b | Detailed description of how the exercise program was progressed | Not reported | - | - | 0 | Not reported |
| 8 | Detailed description of each exercise to enable replication | In the intervention group, physical therapists provided a guidebook to participants, providing information on correct methods to perform the exercises.  For single-leg standing, participants were instructed to maintain a single-leg standing posture for one minute, using light touch on stable desk or chair.  For squats, participants were instructed to move slowly from a standing posture to a half-sitting posture over a 6-second movement time, and then to slowly return to their standing position, with 6 repetitions performed per set.  Heel raises were performed, with 20 repetitions per set. Participants were instructed to complete three full set of exercises per day.  For the walking component of the program, participants were instructed to walk rhythmically, while keeping a correct posture of the head and trunk, for 20–30 minutes per day. | Page 3184 | - | 1 | - |
| 9 | Detailed description of any home program component | The home exercise programs included lower limb training (Locomotion-training). | Page 3184 | - | 1 | - |
| 10 | Describe whether there are any nonexercised components | Not reported | - | - | 0 | Not reported |
| 11 | Describe the type and number of adverse events that occur during exercise | Not reported | - | - | 0 | Not reported |
| 12 | Describe the setting in which exercise are performed | Not reported | - | - | 0 | Not reported |
| 13 | Detailed description of exercise intervention | The following lower limb resistance exercises and balance exercise were used: squats, single-leg standing, and heel raises. Duration of the intervention was 6 months. | Page 3184 | - | 1 | - |
| 14a | Describe whether exercise are generic (one size fits all) or tailored | Not reported | - | - | 0 | Not reported |
| 14b | Detailed description of how exercises are tailored to the individual | Not reported | - | - | 0 | Not reported |
| 15 | Describe the decision rule for determining the starting level | Not reported | - | - | 0 | Not reported |
| 16a | Describe how adherence or fidelity is assessed/measured | Not reported | - | - | 0 | Not reported |
| 16b | Describe the extent to which the intervention was delivered as planned | Despite the convenience of our program, 23.5% of participants in the intervention group (8 out of 34 participants) dropped-out of the study. Furthermore, 16 participants (8%) of the intervention group could not be contacted for baseline evaluation, because the schedule was not matched with theirs. | Page 3187 | - | 1 | - |
| Total score |  |  |  |  | 6 |  |

| Author and year: Bellomo et al., 2013  Title: MUSCLE STRENGTH AND BALANCE TRAINING IN SARCOPENIC ELDERLY: A PILOT STUDY WITH RANDOMIZED CONTROLLED TRIAL  Journal: European Journal of Inflammation  Study location: Italy | | | | | | |
| --- | --- | --- | --- | --- | --- | --- |
| Item | Description | Data extraction-details | Location (pg, URL etc) | | Yes: 1  No:0 | Reasons for rating eg, not reported or not clearly describes |
|  |  |  | Primary paper (Page, table, appendix) | Others (paper, protocol, website, URL) |  |  |
| 1 | Detailed description of the type of exercise equipment | The subject also undertook 20-minute training using the multi-sensory protocol for balance and flexibility with the lmoove system (Allcare Innovations, 26120 Chabeuil, France)  The subjects were invited to perform postural exercises in isometric contractions on a motor platform with elliptic oscillatory movements  Warmup on a stationary bicycle  Subjects received a focused vibratory stimulation for 15 minutes (VISS, VISSMAN S.r.L., Roma, Italy). | Page 195 | - | 1 | - |
| 2 | Detailed description of the qualification, expertise and /or training | Not reported | - | - | 0 | Not reported |
| 3 | Describe whether exercises are performed individually or in a group | Not reported | - | - | 0 | Not reported |
| 4 | Describe whether exercises are supervised or unsupervised; how they are delivered | Supervised | Page 195 | - | 1 | - |
| 5 | Detailed description of how adherence to exercise is measured and reported | Not reported | - | - | 0 | Not reported |
| 6 | Detailed description of motivation strategies | Not reported | - | - | 0 | Not reported |
| 7a | Detailed description of the decision rule(s) for determining exercise progression | Not reported | - | - | 0 | Not reported |
| 7b | Detailed description of how the exercise program was progressed | In weeks 1 to 4, the subjects carried out resistance training on both machines performing 3 sets of 12 repetitions at 60-70% of the FMT, with a 2-minute rest  between sets.  In weeks 5 to 8 weeks, the subjects carried out resistance training on both machines performing 3 sets of 10 repetitions at 75-80% of the FMT, with a rest between  sets of 2 minutes.  In weeks 9 to 12, the subject undertook resistance training on both machines performing 3 sets of 6-8 repetitions at 80% to 85% of the FMT, with a 2-minute  rest between sets | Page 195 | - | 1 | - |
| 8 | Detailed description of each exercise to enable replication | **Global Sensorimotor Training (Gsm)**  The subjects performed 2 sessions per week. A 5-minute cycle ergometer warm-up at an intensity equal to 60% of theoretical maximum heart rate (HRmax) , stretching exercises for the muscles of the lower limbs, and a 5-minute cool down were performed at each training session. The subject also undertook 20-minute training using the multi-sensory protocol for balance and flexibility with the lmoove system (Allcare Innovations, 26120 Chabeuil, France), "Reboost" protocol program according to the manufacturer's parameters. The subjects were invited to perform postural exercises in isometric contractions on a motor platform with elliptic oscillatory movements. Visual feedback required different tasks.  **Resistance Training (Ret)**  The subject performed a resistance training for lower limbs using two isoinertial exercises, leg press and leg extension, 2 sessions for week. The load of the resistance training per each exercise was decided on the percentage of the maximum theoretical force (FMT). At the beginning of each training session, the subject carried out a warmup on a stationary bicycle, pedaling for 10 minutes at an intensity equal to 60% of HRmax, and performed stretching exercises for the muscles of the lower limbs. The subjects performed 1 set of 15 repetitions with a load equal to 30% of FMT as specific warm-up, with the appropriate position on the machine.  **Vibratory mechanical-acousticfocal therapy (Vma)**  Subjects received a focused vibratory stimulation for 15 minutes (VISS, VISSMAN S.r.L., Roma, Italy). The transducer can develop a time-modulated sinusoidal signal *f* up to 300Hz and *p* up to 70 mbar. The size of the transducer was 23.7cm2. The stimulation was applied on the vastus medialis, vastus lateralis and rectus femoris  muscles. The subjects carried out training for 12 weeks: 8 weeks training with 1 session per week, and 3 sessions per week during the last 4 weeks. | Page 195 | - | 1 | - |
| 9 | Detailed description of any home program component | All subjects were asked to keep the same daily habits concerning diet,  social relations and physical activity. | Page 195 | - | 1 | - |
| 10 | Describe whether there are any nonexercised components | Not reported | - | - | 0 | Not reported |
| 11 | Describe the type and number of adverse events that occur during exercise | Not reported | - | - | 0 | Not reported |
| 12 | Describe the setting in which exercise are performed | Not reported | - | - | 0 | Not reported |
| 13 | Detailed description of exercise intervention | **Global Sensorimotor Training (Gsm)**  The subjects performed 2 sessions per week  **Resistance training:**  The subject performed a resistance training for lower limbs using two isoinertial exercises, leg press and leg extension, 2 sessions for week. The subjects performed 1 set of 15 repetitions with a load equal to 30% of FMT as specific warm-up, with the appropriate position on the machine.  **Vibratory mechanical-acousticfocal therapy (Vma)**  The subjects carried out training for 12 weeks: 8 weeks training with 1 session per week, and 3 sessions per week during the last 4 weeks. | Page 195 | - | 1 | - |
| 14a | Describe whether exercise are generic (one size fits all) or tailored | Not reported | - | - | 0 | Not reported |
| 14b | Detailed description of how exercises are tailored to the individual | Not reported | - | - | 0 | Not reported |
| 15 | Describe the decision rule for determining the starting level | The load of the resistance training per each exercise was decided on the percentage of the maximum theoretical force (FMT) | - | - | 1 | - |
| 16a | Describe how adherence or fidelity is assessed/measured | Not reported | - | - | 0 | Not reported |
| 16b | Describe the extent to which the intervention was delivered as planned | Not reported | - | - | 0 | Not reported |
| Total score |  |  |  |  | 7 |  |

| Author and year: Sousa et al., 2013  Title: Is-Once weekly resistance training is enough to prevent sarcopenia?  Journal: Journal of American Geriatric Society  Study location: Portugal | | | | | | |
| --- | --- | --- | --- | --- | --- | --- |
| Item | Description | Data extraction-details | Location (pg, URL etc) | | Yes: 1  No:0 | Reasons for rating eg, not reported or not clearly describes |
|  |  |  | Primary paper (Page, table, appendix) | Others (paper, protocol, website, URL) |  |  |
| 1 | Detailed description of the type of exercise equipment | Not reported | - | - | 0 | Not reported |
| 2 | Detailed description of the qualification, expertise and /or training | Not reported | - | - | 0 | Not reported |
| 3 | Describe whether exercises are performed individually or in a group | Not reported | - | - | 0 | Not reported |
| 4 | Describe whether exercises are supervised or unsupervised; how they are delivered | Supervised | Page 1423 | - | 1 | - |
| 5 | Detailed description of how adherence to exercise is measured and reported | Not reported | - | - | 0 | Not reported |
| 6 | Detailed description of motivation strategies | Not reported | - | - | 0 | Not reported |
| 7a | Detailed description of the decision rule(s) for determining exercise progression | Not reported | - | - | 0 | Not reported |
| 7b | Detailed description of how the exercise program was progressed | Not reported | - | - | 0 | Not reported |
| 8 | Detailed description of each exercise to enable replication | The RT consisted of seven exercises (bench press, leg press, latissimus dorsi pull-down, leg extension, military press, leg curl, and arm curl) | Page 1423 | - | 1 | - |
| 9 | Detailed description of any home program component | Not reported | - | - | 0 | Not reported |
| 10 | Describe whether there are any nonexercised components | Not reported | - | - | 0 | Not reported |
| 11 | Describe the type and number of adverse events that occur during exercise | Not reported | - | - | 0 | Not reported |
| 12 | Describe the setting in which exercise are performed | Not reported | - | - | 0 | Not reported |
| 13 | Detailed description of exercise intervention | The resistance training were performed 1 day per week for 32 weeks at 65% to 75% of 1-RM (three sets, 8–12 repetitions). | Page 1423 | - | 1 | - |
| 14a | Describe whether exercise are generic (one size fits all) or tailored | Not reported | - | - | 0 | Not reported |
| 14b | Detailed description of how exercises are tailored to the individual | Not reported | - | - | 0 | Not reported |
| 15 | Describe the decision rule for determining the starting level | Muscle strength was measured using the 30-second chair-stand and arm-curl tests, and maximum strength was measured (in the RTG) using the one-repetition maximum (1-RM) method. | Page 1423 | - | 1 | - |
| 16a | Describe how adherence or fidelity is assessed/measured | Not reported | - | - | 0 | Not reported |
| 16b | Describe the extent to which the intervention was delivered as planned | Not reported | - | - | 0 | Not reported |
| Total score |  |  |  |  | 4 |  |

| Author and year: Kim et al., 2012  Title: Effects of exercise and amino acid supplementation on Body composition and Physical function in community dwelling elderly Japanese sarcopenic women: A RCT  Journal: Journal of American Geriatric society  Study location: Japan | | | | | | |
| --- | --- | --- | --- | --- | --- | --- |
| Item | Description | Data extraction-details | Location (pg, URL etc) | | Yes: 1  No:0 | Reasons for rating eg, not reported or not clearly describes |
|  |  |  | Primary paper (Page, table, appendix) | Others (paper, protocol, website, URL) |  |  |
| 1 | Detailed description of the type of exercise equipment | Ankle weights, resistance band | Page 19 | - | 1 | - |
| 2 | Detailed description of the qualification, expertise and /or training | Exercise instructor and assistant trainer but qualification not reported | Page 19 | - | 1 | - |
| 3 | Describe whether exercises are performed individually or in a group | Each exercise intervention group was divided into two subgroups, with participants exercising together within their assigned group in one of four exercise sessions  offered per day | Page 19 | - | 1 | - |
| 4 | Describe whether exercises are supervised or unsupervised; how they are delivered | Supervised but how it was delivered not clearly described | Page 19 | - | 1 | - |
| 5 | Detailed description of how adherence to exercise is measured and reported | Not reported | - | - | 0 | Not reported |
| 6 | Detailed description of motivation strategies | Not reported | - | - | 0 | Not reported |
| 7a | Detailed description of the decision rule(s) for determining exercise progression | The principal investigator, along with the exercise instructor and assistant trainers, assessed each individual’s ability to increase intensity | Page 19 | - | 1 | - |
| 7b | Detailed description of how the exercise program was progressed | When the exercises were properly executed without significant fatigue or loss of proper execution, the resistance was increased. The progressive resistance was provided through the use of resistance bands or ankle weights. Intensity was maintained at approximately 12 to 14 on the Borg Rate of Perceived Exertion scale. | Page 19 | - | 1 | - |
| 8 | Detailed description of each exercise to enable replication | **Chair exercise:**  The chair-seated exercises were used in the early stages of the program because the participants were frail older adults, and it provided a secure and stable position. Repetitions of toe raises, heel raises, knee lifts, knee extensions, and others were performed while seated on a chair. Hip flexions, lateral leg raises, and repetitions  of other exercises were performed standing upright behind the chair and holding the back of the chair for stability.  **Ankle-weight exercise:**  To strengthen lower extremities, a fixed weight was placed on the ankle while participants performed strengthening exercises. Weights of 0.50, 0.75, 1.00, and 1.50 kg were prepared and used in accordance with each participant’s strength level as the  resistance progressively increased. The exercises performed using these ankle weights included seated knee flexion and extension and standing knee flexion and extensions.  **Exercises using a resistance band:**  Resistance bands were used to strengthen the upper and lower body. Lower body exercises included leg extension and hip flexion. Upper body exercises included double-arm pull downs and biceps curls.  **Balance and gait training:**  The balance training was focused on improvement of static, dynamic, and lateral balancing ability. Exercises included standing on one leg, multidirectional weight shifts, tandem stand, and tandem walk. Participants practiced proper gait mechanics that focused on the maintenance of stability during walking and increasing stride length, toe elevation of the forward limb, heel elevation of the rear limb, frequency of stepping, and heel–floor angle. Exercises included raising the toes (dorsiflexion) during the forward swing of the leg, kicking off the floor with the ball of the foot, walking with directional changes, and gait pattern variations. | Page 19 | - | 1 | - |
| 9 | Detailed description of any home program component | Not reported | - | - | 0 | Not reported |
| 10 | Describe whether there are any nonexercised components | Essential amino acid supplementation (AAS) was provided for the participants in the AAS groups every 2 weeks. Packets of powdered amino acid supplements (42.0% leucine, 14.0% lysine, 10.5% valine, 10.5% isoleucine, 10.5% threonine, 7.0% phenylalanine, and 5.5% other) were provided for the participants to be taken with water or milk, and they were instructed to take the 3-g supplement two times a day (6 g  daily) every day for 3 months | Page 19 | - | 1 | - |
| 11 | Describe the type and number of adverse events that occur during exercise | Not reported | - | - | 0 | Not reported |
| 12 | Describe the setting in which exercise are performed | Tokyo Metropolitan Institute of Gerontology (TMIG) | Page 19 | - | 1 | - |
| 13 | Detailed description of exercise intervention | A comprehensive physical fitness and muscle mass enhancement training program of moderate intensity was provided for the participants in the exercise groups. The exercise intervention consisted of 60-minute exercise sessions held at the TMIG twice per week for 3 months. Each exercise session consisted of a 5-minute warmup, 30 minutes of strengthening exercise, 20 minutes of balance and gait training, and 5 minutes of cool down. The strengthening exercises were performed in a progressive sequence from seated to standing positions. For each type of exercise, participants were instructed to complete up to eight repetitions of the movements. | Page 19 | - | 1 | - |
| 14a | Describe whether exercise are generic (one size fits all) or tailored | Not clearly described | - | - | 0 | Not clearly described |
| 14b | Detailed description of how exercises are tailored to the individual | Not reported | - | - | 0 | Not reported |
| 15 | Describe the decision rule for determining the starting level | Not reported | - | - | 0 | Not reported |
| 16a | Describe how adherence or fidelity is assessed/measured | Not reported | - | - | 0 | Not reported |
| 16b | Describe the extent to which the intervention was delivered as planned | Eleven participants (exercise + AAS = 4, exercise = 3, AAS = 2, Health education [HE] = 2) were unable to complete the study after randomization because of spouse care (n = 3), admission to nursing home (n = 2), lack of motivation (n = 2), severe knee or back pain (n = 1), death (n = 1), falls and hip fracture (n = 1), and hospitalization (n = 1). | Page 20 | - | 1 | - |
| Total score |  |  |  |  | 11 |  |
