# Supplementary material for: A review of the components of exercise prescription for sarcopenic older adults
Source: Eur Geriatr Med. 2022 Sep 2;13(6):1245–80. doi: 10.1007/s41999-022-00693-7 (PMC9722805; doi:10.1007/s41999-022-00693-7)
Supplement: Supplementary file 2 — Supplementary file2 (PDF 69 kb) [file 41999_2022_693_MOESM2_ESM.pdf]

PubMed search strategy

#1 (((((“sarcopeni*) OR (“muscle mass loss”)) OR (muscle atrophy”)) OR (“muscle weakness”)) OR (“muscle loss”)) OR (“reduced skeletal muscle mass”)

#2 (((((“aged”) OR (“older people”)) OR (“older adult”)) OR (“elderly”)) OR (“senior citizens”)) OR (“elderly people”)

#3 ((((((((((“physical therapy”) OR (“therapeutic exercise”)) OR (“rehabilitation programme”)) OR (“multicomponent intervention”)) OR (“resistance training”)) OR (“balance exercise”)) OR (“exercise”)) OR (“aerobic exercise”)) OR (“flexibility exercise”)) OR (“endurance training”)) OR (“physiotherapy”)

#4 #1 AND #2 AND #3

No filters applied

Scopus search strategy

1 “sarcopenia” OR “sarcopenic” OR “muscle mass loss” OR “muscle atrophy” OR “muscle weakness”

2 “aged” OR “older adult” OR “ older people” OR “elderly” OR “old age”

3 “physiotherapy” OR “physical therapy” OR “multicomponent intervention” OR “reablement programme” OR “resistance training” OR “balance training” OR “aerobic training” OR “cognitive training”

4 “residential home settings” OR “residential care settings” OR “residential care facility” OR “old age homes” OR “long term care facility”

5 (“sarcopenia” OR “sarcopenic” OR “muscle mass loss” OR “muscle atrophy” OR “muscle weakness”) AND (“aged” OR “older adult” OR “ older people” OR “elderly” OR “old age”) AND (“physiotherapy” OR “physical therapy” OR “multicomponent intervention” OR “reablement programme” OR “resistance training” OR “balance training” OR “aerobic training” OR “cognitive training”) AND (“residential home settings” OR “residential care settings” OR “residential care facility” OR “old age homes” OR “long term care facility”)

No restrictions applied

Web of Science search strategy

1 (((((ALL=(“sarcopenia”)) OR ALL=(“sarcopenic”)) OR ALL=(“reduced skeletal muscle mass”)) OR ALL=(“muscle mass loss”)) OR ALL=(“muscle atrophy”)) OR ALL=(“muscle weakness”)

2 ((((ALL=(“aged”)) OR ALL=(“older adult”)) OR ALL=(“older people”)) OR ALL=(“elderly”)) OR ALL=(“older person”)

3 (((((((((ALL=(“physiotherapy”)) OR ALL=(“physical therapy”)) OR ALL=(“reablement programme”)) OR ALL=(“resistance exercise”)) OR ALL=(“multicomponent exercise”)) OR ALL=(“aerobic training”)) OR ALL=(“cognitive exercise”)) OR ALL=(“therapeutic exercise”)) OR ALL=(“balance exercise”)) OR ALL=(“exercise”)

4 #1 AND #2 AND #3

No filters applied

CINAHL search strategy

S1 sarcopenia OR sarcopenia OR muscle weakness OR muscle atrophy OR muscle loss

S2 old age OR elderly OR aged people OR senior OR older people OR older adults

S3 physiotherapy OR physical therapy OR rehabilitation OR exercise OR intervention

S4 reablement

S5 multicomponent exercise training

S6 long-term care OR nursing home OR residential care OR assisted living

S7 (sarcopenia OR sarcopenia OR muscle weakness OR muscle atrophy OR muscle loss) AND (old age OR elderly OR aged people OR senior OR older people OR older adults) AND (physiotherapy OR physical therapy OR rehabilitation OR exercise OR intervention) OR reablement OR multicomponent exercise training AND (long-term care OR nursing home OR residential care OR assisted living)

No restrictions applied

Embase search strategy

#1 “sarcopenia” OR “muscle atrophy” OR “muscle mass” OR “muscle weakness”

#2 “aged” OR “older adult” OR “senior citizen”

#3 “physiotherapy” OR “multicomponent intervention” OR “resistance training” OR “balance training”

#4 #1 AND #2 AND #3

No filters applied
